# Fat Replacers in Frozen Desserts: Functions, Challenges, and Strategies

**DOI:** 10.1111/1541-4337.70191

**Published:** 2025-05-15

**Authors:** Zhaoyi Tang, Shuyue Yang, Weitian Li, Jinhui Chang

**Affiliations:** ^1^ Department of Food Science and Nutrition The Hong Kong Polytechnic University Hong Kong China; ^2^ The Hong Kong Polytechnic University Jinjiang Technology and Innovation Research Institute Quanzhou China; ^3^ Research Institute for Future Food The Hong Kong Polytechnic University Hong Kong China; ^4^ Bo InnoHealth Biotechnology Company Limited, Hong Kong Science Park Hong Kong China

**Keywords:** fat replacers, frozen desserts, frozen yogurt, ice cream, physicochemical properties, reduced‐fat foods, sensory properties

## Abstract

Frozen desserts are highly valued for their creamy texture and rich mouthfeel, primarily due to their high‐fat content. However, the increasing consumption of these products has raised concerns regarding excessive fat intake, which has been linked to health issues such as obesity, diabetes, and cardiovascular disease (CVD). Therefore, there is growing interest in developing fat replacers. Fat replacers can mimic the physicochemical and sensory properties of natural fats in frozen desserts, including texture, mouthfeel, and flavor interaction, providing a comparable experience with a reduced calorie content. However, fat reduction in frozen desserts often leads to undesirable changes, including reduced smoothness and creaminess, increased chalkiness, and the emergence of dark colors and off‐flavors. To mitigate these challenges, various strategies have been explored, including optimizing the ratio of ingredients, incorporating masking flavors, modifying processing techniques, and blending with stabilizers. While existing reviews highlight the benefits of fat replacers, they often focus on limited frozen dessert types and provide insufficient insight into replacement mechanisms and improvement strategies. This review aims to bridge this gap by examining a wide range of frozen desserts, comprehensively analyzing protein‐based, carbohydrate‐based, lipid‐based, and complex fat replacers, and detailing their mechanisms of action, application challenges, and effects on the final product quality. Additionally, strategies for enhancing the sensory attributes of reduced‐fat frozen desserts and future directions are discussed, ultimately supporting the development of sustainable, healthier, and consumer‐acceptable fat alternatives in the food industry.

## 1 Introduction

Frozen desserts are widely favored for their ease of consumption and distinctive palatability, making them a refreshing and enjoyable choice, especially during hot weather (Devaraj 2015). The global frozen dessert market is experiencing significant growth, with its value projected to grow from USD 120.00 billion (2025) to USD 196.30 billion (2035) at a compound annual growth rate (CAGR) of 6.1% (Future Market Insights 2025). One of the key trends observed in this market is the increasing preference for low‐fat and low‐calorie frozen desserts (Sipple et al. 2022; Gomes Da Cruz et al. 2025).

Frozen desserts are complex food systems consisting of ice crystals, air cells, and partially coalesced fat droplets entrapped in a continuous freeze‐concentrated aqueous (serum) phase containing water, proteins, and other soluble components, such as sweeteners and stabilizers (Soukoulis et al. 2014; VanWees et al. 2022). Examples of frozen desserts include ice cream, gelato, frozen yogurt, ice milk, milkshakes, sherbets, and frozen mousse (Duquenne et al. 2016; Goff 2022), with fat contents typically ranging from 0% to 18% (Goff 2022). Notably, in conventional cow milk, saturated fats comprise approximately 70% of the fat content by weight (Lindmark Månsson 2008). These saturated fats promote fat crystallization by forming dense fat crystal networks, which increases the rigidity of the network structure, reduces the melting rate, and ultimately improves ice cream quality (Monié et al. 2023; M. Zhao et al. 2023).

However, excessive fat consumption, particularly saturated and trans fats, is associated with increased cardiovascular disease (CVD) mortality (Son et al. 2024), including coronary heart disease mortality (Reynolds 2022; Aramburu et al. 2024). Additionally, saturated fats have been linked to an increased unhealthy proinflammatory effect (Ma et al. 2025) and an increased risk of cancer mortality (Y. Ma et al. 2024), such as breast cancer (Aramburu et al. 2024). More critically, trans fats are associated with higher all‐cause mortality (Kim et al. 2021). In response to these health concerns, there is a growing interest in developing healthier products with reduced fat and calorie content (Sipple et al. 2022), leading to the development of fat replacers that are essential for the development of reduced‐calorie bakery (Yazar and Rosell 2023), confectionery (Hadnađev et al. 2011), meat (Paglarini et al. 2022), and frozen dessert products (Akbari et al. 2019), which preserve their sensory and functional qualities while reducing traditional fats (Zoulias et al. 2002; Saeed et al. 2023).

In frozen desserts, fat replacers can enhance their quality by improving their mouthfeel, water holding capacity (WHC), flavor retention, and structural stability (Akbari et al. 2019), but sometimes may introduce challenges. In physiochemical aspect, reducing fat content disrupts the network of fat globules, resulting in brittleness, iciness, coarseness, and shrinkage of the final product (Akbari et al. 2019; Hossain et al. 2021). In sensory aspect, some protein‐ or carbohydrate‐based fat replacers also introduce undesirable sensory changes, including off‐flavors, bitterness, and darkened color (Friedeck et al. 2003; Bayarri et al. 2010; de Moraes Crizel et al. 2013; Li et al. 2015; Guler‐Akin et al. 2021). Minimizing side effects remains a growing research direction for fat replacers. Unlike prior reviews that primarily concentrated on limited fat replacers without addressing the associated challenges and strategies, this work aims to provide a comprehensive theoretical foundation for the research and development of reduced‐fat frozen desserts by exploring the functions and challenges of four types of distinct fat replacers and proposing strategies to improve their overall quality. Future perspectives on the development of fat replacers in frozen desserts are also provided, highlighting the directions of innovation in this field.

## 2 Effect of Fat on the Physicochemical and Sensory Properties of Frozen Desserts

Fat is a general term that represents oils and lipids and comprises various organic compounds, including fatty acids, acylglycerols, and phospholipids (Ratnayake and Galli 2009). In addition to being an important macronutrient and energy source for the human body (German 2011), fat is essential for ensuring optimal physicochemical and sensory properties of frozen desserts, contributing to attributes such as structure, texture, flavor, mouthfeel, and appearance. Although consumers desire a significant reduction in fat content in reduced‐fat foods, fat can hardly be removed owing to its essential roles.

### 2.1 Effect of Fat on the Physicochemical Properties of Frozen Desserts

Frozen desserts typically follow similar production processes involving eight key stages: ingredient mixing, pasteurization, homogenization, aging, flavoring and coloring, aeration and freezing, addition of fruits and nuts, and packaging and hardening (Goff 2002; Dowlati et al. 2017). After aging, milk proteins are displaced from the surface of fat globules (Hartel et al. 2017; Y. Zhao et al. 2023). Fat globules partially coalesce during intense shear forces in the freezing stage and further assemble into a fat globule network (Goff 2002). This fat globule network is crucial for preserving structural integrity and preventing phase separation between water and air (Munk et al. 2018), while the absence of this network can result in a macroscopically phase‐separated system of foam and liquid upon thawing, associated with unfavorable texture and mouthfeel (Amador et al. 2017; Munk et al. 2018). The network further contributes to other desirable attributes, including dryness, coldness, and melting rate (Koxholt et al. 2001; Javidi et al. 2016). In summary, fat plays a fundamental role in frozen desserts by ensuring structural stability, enhancing texture, regulating melting behavior, and hence achieving optimal physicochemical properties of frozen desserts.

### 2.2 Effect of Fat on the Sensory Properties of Frozen Desserts

Fat also plays several key roles in shaping the sensory properties of frozen desserts (Table 1). First, it facilitates flavor development by controlling the release of flavorful volatile compounds that contribute to complex flavor profiles (Chung et al. 2003; Akbari et al. 2019). In addition, fat enhances the perception of creaminess and thickness by reducing friction during oral processing and by increasing viscosity (Adapa et al. 2000; Dresselhuis et al. 2008). Furthermore, specific types of fat, such as palm midfraction, have been shown to improve the visual attributes of ice cream by increasing brightness and diminishing undesirable redness and yellowness (Nazaruddin et al. 2008).

**TABLE 1:** Fat content in frozen desserts and physicochemical and sensory functions of fat.

Frozen desserts	Fat content (%)	Physicochemical functions	Sensory functions	References
Ice cream	Low fat: 2–5 Standard (Full fat): 10–12 Super premium: 14–18	Carry flavor compounds, stabilize air bubbles, and contribute to the formation of small ice crystals	Provide smooth and rich texture, enhance flavor	(Bowen et al. 2003; Goff 2022)
Gelato	4–8	Contribute to desirable food structure and texture, protect the mouth for easy oral processing	Enrich flavor, provide a rich and creamy mouthfeel, and enhance overall sensory experience	(Shingh et al. 2020)
Frozen yogurt	Nonfat: <0.5 Regular: 3.25–6	Contribute to the formation of a stable emulsion, enhance viscosity, and stabilize the structure of the product	Promote creaminess, smoothness, and desirable mouthfeel while reducing grittiness and enhancing flavor perception	(Inoue et al. 1998; Ordonez et al. 2000; Skryplonek et al. 2017; Goff 2022)
Ice milk	3–5	Stabilize emulsion, enhance air incorporation, and ensure ideal freezing properties	Contribute to the creamy texture and smoothness, and improve melting qualities	(Schmidt et al. 1993; Goff 2022)
Milkshakes	3	Stabilize emulsion, prevent ice crystal formation, and enhance air incorporation during whipping	Contribute to creamy mouthfeel, improve flavor release, and deliver richness	(Bowen et al. 2003; Dutta et al. 2021)
Sherbets	1–2	Lubricate freezer blades during dynamic freezing; reduce ice crystal growth through limited fat globule stabilization	Enhance mild creaminess and mouth‐coating perception despite low fat content; support subtle flavor delivery	(Marshall et al. 2003; Goff and Hartel 2013)

Table 1 provides a detailed summary of the varying fat contents and specific physicochemical and sensory functions of fats in different types of frozen desserts, based on the existing literature. For example, in ice cream, fat contributes to the formation of small ice crystals and the stabilization of air bubbles, which are essential for a smooth and rich texture (Bowen et al. 2003; Goff 2022). In gelato, fat enriches the flavor and provides a rich and creamy mouthfeel (Shingh et al. 2020). In frozen yogurt, fat stabilizes the emulsion and promotes a desirable mouthfeel (Inoue et al. 1998; Ordonez et al. 2000; Skryplonek et al. 2017; Goff 2022), whereas in ice milk, it enhances the creamy texture and smoothness (Schmidt et al. 1993; Goff 2022). These examples illustrate the critical role of fat in maintaining structural integrity, enhancing texture, and improving the overall sensory experience of various frozen desserts.

## 3 Functions of Fat Replacers in Frozen Desserts

**FIGURE 1:**
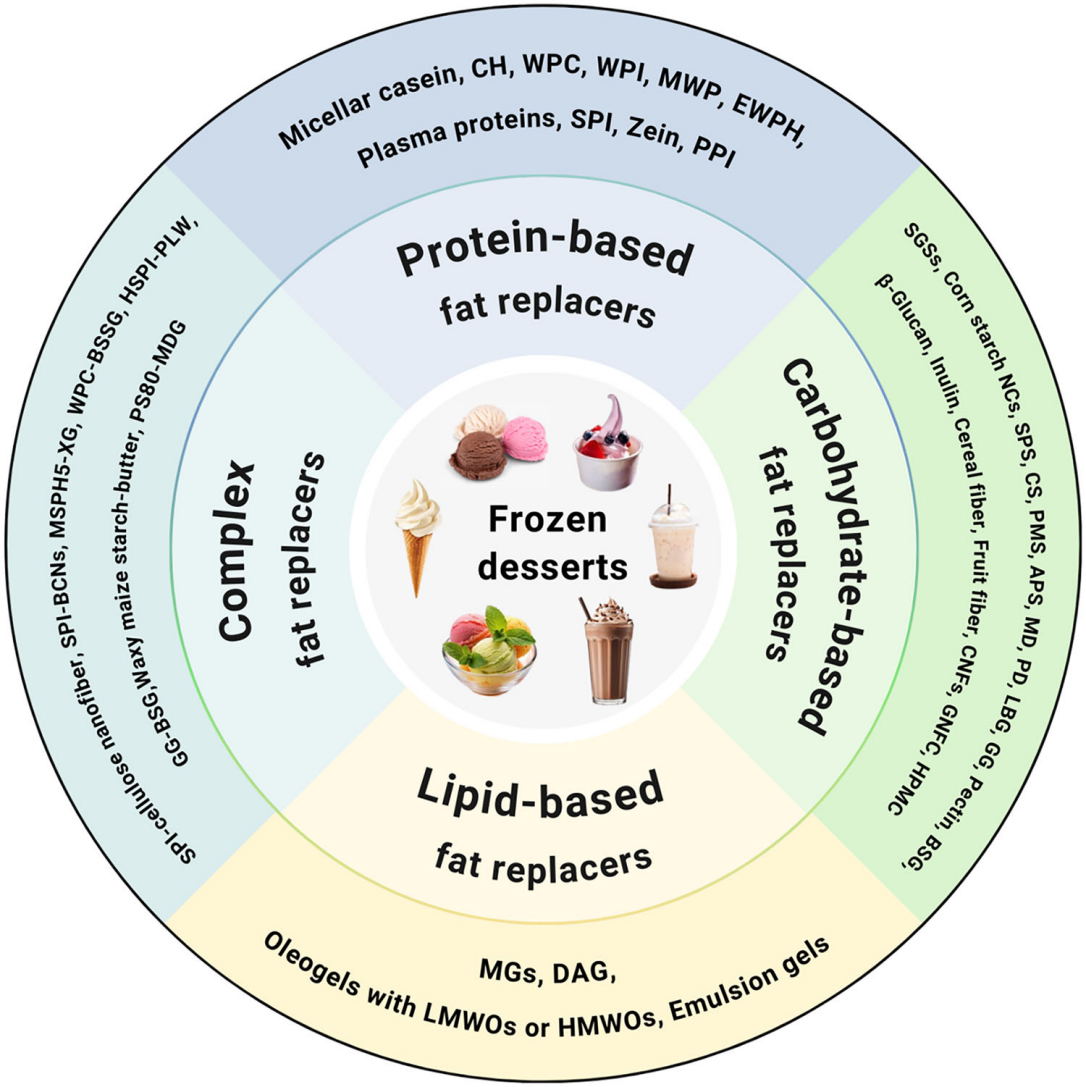
Classification of fat replacers based on their composition. *Source*: Created with BioRender.com.

According to U.S. regulations, foods labeled as reduced‐fat are defined as those containing at least 25% less fat than their full‐fat counterparts (Code of Federal Regulations 2025). The primary goal of developing fat replacers is to reduce the caloric content and/or saturated fat content while preserving their quality and safety (Gao et al. 2024). An ideal fat replacer should be safe, low in cost and calories, and capable of maintaining the sensory attributes of natural fats, including texture, flavor, and overall eating quality (Sun and Fang 2021). It should also be stable during storage and processing and should not interfere with the nutritional value or absorption of other food components (Gao et al. 2024). The replacement mechanisms of fat replacers in frozen desserts vary according to their compositions. By categorizing fat replacers into protein‐, carbohydrate‐, lipid‐based, and complex types (Figure 1), their unique contributions to reduced‐fat frozen desserts can be better understood.

### 3.1 Protein‐Based Fat Replacers

**FIGURE 2:**
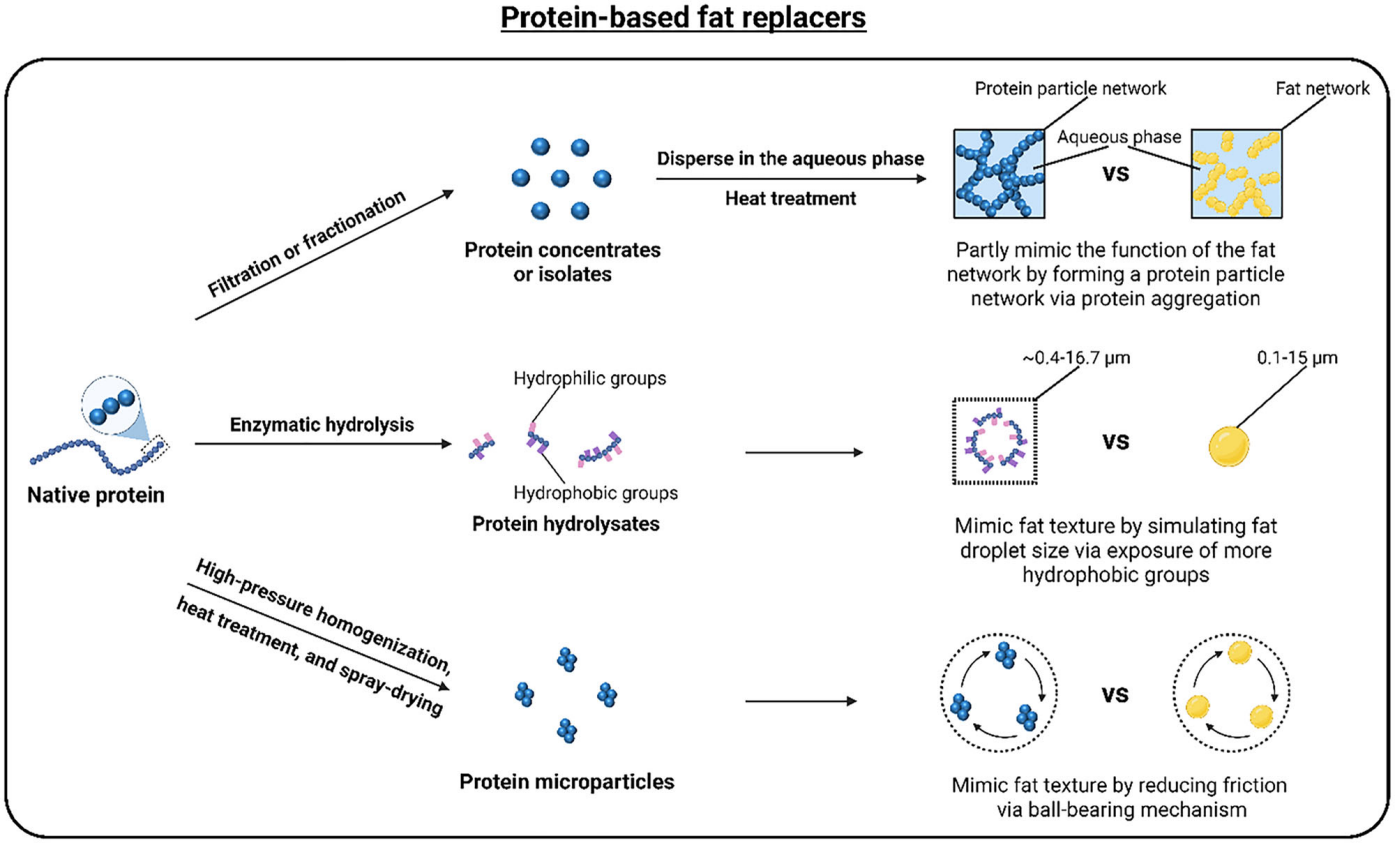
Schematic showing the mechanism of protein‐based fat replacers in frozen desserts compared to their full‐fat counterparts. *Source*: Created with BioRender.com.

Currently, protein‐based fat replacers can be derived from animal, plant, or microbial sources and can be classified into protein concentrates or isolates, hydrolysates, and microparticles. Animal and plant proteins are widely used as fat replacers in frozen desserts (Table 2). Animal proteins, particularly whey proteins, have been extensively studied as fat replacers in frozen desserts (Salama et al. 2025). Additionally, plant proteins are increasingly being developed as fat replacers because they are more environment‐friendly and economical (Gomes and Sobral 2022; Munialo and Vriesekoop 2023) and are often more cost‐effective and widely available than animal proteins (Hertzler et al. 2020). Protein‐based fat replacers in frozen desserts function through three distinct mechanisms to mimic the texture and characteristics of their full‐fat counterparts (Figure 2). First, protein concentrates or isolates, obtained through filtration or fractionation (Nourmohammadi et al. 2023), are dispersed in the aqueous phase and heated to form protein aggregates (Akalın et al. 2008). As these aggregates increase, they develop into larger, nondispersible agglomerates. At sufficient concentrations, these agglomerates can form a protein network that partially mimics the functionality of the fat network during melting (X. Liu et al. 2024). Second, enzymatic hydrolysis of native proteins generates protein hydrolysates that expose more hydrophobic groups (Kiewiet et al. 2018; Chen et al. 2019; Zang et al. 2019; Nourmohammadi et al. 2023; Gao et al. 2024; Patil et al. 2024). These hydrophobic groups allow the hydrolysates to mimic fat texture by adjusting their droplet size to 0.4–16.7 µm (Cui et al. 2013), similar to the droplet size (0.1–15 µm) of milk fat globules (MFGs) (Truong et al. 2016). Third, protein microparticles produced through high‐pressure homogenization, heat treatment, and spray‐drying can reduce friction by acting as a ball‐bearing mechanism. This mechanism enhances the mouthfeel and texture of frozen desserts by mimicking the lubricating effect of fat (Liu et al. 2016; Nourmohammadi et al. 2023; Gao et al. 2024). Collectively, these protein‐based fat replacers provide multiple strategies to achieve fat‐like properties while reducing the calorie content of frozen desserts (Table 2).

**TABLE 2:** Applications of protein‐based fat replacers in frozen desserts.

Types	Sources	Forms	Applications	Suggested replacement ratio (%)	Functions	References
Animal protein‐based	Casein	Micellar casein	Milk ice cream	≥67 (with 5%–6% micellar casein)	Enhance overall protein content and improve sensory properties	(Polishchuk et al. 2020)
		Casein hydrolysate (CH)	Camel milk ice cream	74 (with 2% CH)	Improve flavor, odor, and foam‐forming ability, increase viscosity, decrease melting rate	(Hajian et al. 2020)
	Whey protein	Whey protein concentrate (WPC)	Vanilla ice cream	20–40	Decrease viscosity, achieve overrun, melting rate, and sensory qualities comparable to the full‐fat control	(Khillari et al. 2007)
		WPC with high hydrostatic pressure (HHP) treatment	Vanilla ice cream	≈54 (with 10% HHP‐treated WPC)	Increase hardness, improve overrun and foam stability	(Lim et al. 2008)
		Whey protein isolate (WPI)	Milk ice cream	40, 70 (with 4% WPI)	Increase apparent viscosity, consistency coefficient, and hardness, decrease flow behavior index and melting rate	(Akalın et al. 2008)
		WPI with transglutaminase (TGase) treatment	Milk ice cream	50 (with 4 g/L TGase‐treated WPI)	Enhance color, overrun, and texture, increase viscosity, reduce hardness	(Danesh et al. 2017)
		Microparticulated whey protein (MWP)	Ice milk	≤58 (with 3.4% Simplesse^®^ 100)	Increase air incorporation, achieve rheological and melting properties comparable to the full‐fat control	(Schmidt et al. 1993)
Animal protein‐based	Whey protein	Microparticulated whey protein (MWP)	Vanilla ice cream	≈91 (with 4.8% Dairy‐Lo™)	Provide excellent gumminess, increase viscosity, decrease melting rate	(Ohmes et al. 1998)
				50 (with 6% Simplesse^®^ 100)	Provide firmness and sensory properties similar to the full‐fat control	(Yilsay et al. 2006)
	Egg white proteins (EWPs)	Egg white protein hydrolysate (EWPH)	Milk ice cream	≈100	Decrease hardness, improve texture, maintain sensory qualities similar to the full‐fat control	(López‐Martínez et al. 2021)
	Plasma proteins	Plasma powder from porcine, bovine, or ovine blood	Chocolate ice cream	≤30	Increase hardness and lightness	(Csurka et al. 2023)
Plant protein‐based	Soy protein	Soy protein isolate (SPI)	Vanilla ice cream	≈100 (with 4% medium‐sized SPI of approximately 4 µm)	Provide creaminess, dense texture, and good lubrication behavior, improve mouth coating, decrease melting rate, achieve sensory qualities similar to the full‐fat control	(X. Liu et al. 2024)
Plant protein‐based	Zein	Alcalase hydrolyzed zein	Milk ice cream	10	Increase water content, promote soft gel formation, improve emulsion stability, reduce lipid oxidation, shear stress, and viscosity	(Zhang et al. 2023)
	Pea protein	Pea protein isolate (PPI)	Vanilla ice cream	≈60 (with 6% or 12% PPI)	Increase viscosity, hardness, adhesiveness, springiness, cohesiveness, and gumminess, decrease overrun and melting rate	(Guler‐Akin et al. 2021)

#### 3.1.1 Animal Proteins

Among animal proteins, milk, egg white, and plasma proteins have been studied as fat replacers in frozen desserts (Table 2). These proteins offer unique functional properties, such as gelation, WHC, and emulsification (Qian 2009; Floret et al. 2023), which enable them to mimic the physicochemical properties and sensory attributes of fat while providing fewer calories (Abbas et al. 2024). Various fabrication methods, such as thermal‐mechanical treatment, antisolvent precipitation, and enzymatic hydrolysis, have been employed to create protein‐based fat replacers (Nourmohammadi et al. 2023). However, consumer acceptance of these replacers is limited because of differences in taste and texture compared to traditional animal fats (Munialo and Vriesekoop 2023). Therefore, the effects of processing, particularly protein denaturation, on the functional and structural characteristics of protein‐based fat replacers require further investigation (Yashini et al. 2021; Munialo et al. 2022).

##### 3.1.1.1 Milk Proteins

Milk proteins, primarily casein and whey proteins, are integral components of bovine milk, accounting for 3.0%–3.5% of its composition (Goulding et al. 2020). Casein constitutes approximately 80% of the total protein content of milk, whereas whey proteins constitute the other 20%. Milk proteins exhibit diverse functional properties, including emulsification, foaming, and gelation (Akbari et al. 2019), which are crucial for their application as fat replacers in frozen desserts such as ice cream. However, the functions of milk proteins can be affected by processing conditions, such as pH and temperature. Therefore, careful optimization is required to achieve the desired textural and sensory attributes of reduced‐fat frozen desserts.

Casein, a major milk protein, exists in a micellar structure that can be isolated by the microfiltration of skim milk (Hammam et al. 2021; Xia et al. 2022). Micellar casein has unique physicochemical properties, including thermal stability, solubility, and water‐binding ability (Holt et al. 2013). Polishchuk et al. (2020) used response surface methodology (RSM) to determine the optimal content of micellar casein in ice creams with varying fat contents (0%–15%). Based on their mathematical models, 5%–6% micellar casein and 8.7%–9.7% total protein content were needed for ice cream with 0%–5% fat content to achieve the maximum key quality parameters, including melt resistance, overrun, and organoleptic properties. However, challenges in using micellar casein as a fat replacer include alterations in product texture and potential off‐flavors. For instance, in the production of low‐fat Cheddar cheese, micellar casein concentrate (MCC) was used to replace fat, resulting in a softer texture and the introduction of bitter and grape‐tortilla off‐flavors (Amelia et al. 2013). Although this study focused on cheese, similar problems may occur in frozen desserts. The hydrophobic nature of casein micelles can disrupt the stability of emulsions in frozen desserts (Yan et al. 2024), while the exposure of hydrophobic amino acid residues (particularly proline‐rich sequences) can impart detectable bitterness and astringency (Aluko 2017). Additionally, the structure and integrity of casein micelles are influenced by factors such as pH, temperature, and ionic environment (Liao et al. 2022; Khan et al. 2024). In summary, although micellar casein offers promising benefits as a fat replacer in frozen desserts, its application requires careful consideration to mitigate the challenges associated with texture and flavor.

Whey proteins, including whey protein isolate (WPI), whey protein concentrate (WPC), and microparticulated whey protein (MWP), are increasingly used as fat replacers in frozen desserts because of their functional properties (Table 2). WPC is produced by ultrafiltration of whey, retaining a protein content of 34%–80% (Schmidmeier et al. 2019), and is known for its emulsifying and water‐binding capabilities, making it suitable for partial fat replacement in frozen desserts. Khillari et al. (2007) discovered that incorporating WPC up to 40% as a fat replacer in ice cream did not significantly compromise sensory qualities and physicochemical properties, recommending 20%–40% fat replacement for acceptable low‐fat ice cream production. WPI, with a protein content of over 90%, is obtained by further processing WPC to remove fat and lactose (Carter and Drake 2018). It offers a purer protein source with enhanced gelling and foaming properties, which are beneficial for maintaining the texture and mouthfeel of reduced‐fat frozen desserts. Akalın et al. (2008) investigated the rheological properties of ice cream with 4% WPI as a fat replacer in reduced‐fat (6%) and low‐fat (3%) formulations and found significant increases in apparent viscosity, consistency index, and hardness compared to that of full‐fat (10%) ice cream. This study also reported that WPI improved melting resistance more than inulin (another fat replacer used in this study). MWP is created by heat‐induced denaturation and aggregation of whey proteins, forming microparticles that mimic the creamy texture of fat (Ipsen 2017). This allows for significant fat reduction while maintaining the desirable sensory attributes of frozen desserts. Yilsay et al. (2006) found that using commercial MWP (Simplesse^®^ 100) as a fat replacer at 6% of the total mix in low‐fat (6%) and fat‐free (0.5%) vanilla ice creams improved the texture and sensory characteristics without affecting the vanillin flavor. This study demonstrated that the addition of Simplesse^®^ 100 increased viscosity and provided a texture similar to that of full‐fat (12%) ice cream. In summary, the functions of whey proteins make them promising fat replacers for developing healthier frozen desserts with reduced fat content.

##### 3.1.1.2 Egg White Proteins (EWPs)

EWPs are valuable food ingredients in the food industry because of their functional properties, including gelation, foaming, and emulsification (Razi et al. 2023). However, EWPs are prone to heat‐induced denaturation, with a denaturation temperature range of approximately 60–68°C, which can lead to gelation and aggregation, thereby limiting their application in processes that involve heat (Alavi et al. 2020). This thermal sensitivity necessitates the exploration of alternative methods to enhance stability and functionality. One promising approach is the enzymatic hydrolysis of EWPs, which results in egg white protein hydrolysates (EWPH). Moderate hydrolysis by proteases can improve the absorption rate and nutritional value of EWPs while enhancing their functional properties, such as solubility, foaming, emulsification, and WHC (Pokora et al. 2013; Lyu et al. 2023), making them suitable fat replacers in the preparation of frozen desserts. For instance, López‐Martínez et al. (2021) explored the potential of EWPH as a fat replacer in ice cream and demonstrated that EWPH‐based ice cream could serve as a healthy alternative for consumers with lactose intolerance, milk allergies, or those seeking low‐fat and low‐sugar options. However, challenges remain in optimizing EWPH for specific frozen desserts because its functionality can be affected by factors such as the degree of hydrolysis, enzyme type, and processing conditions (Wouters et al. 2016). Taken together, although further optimization is needed to tailor its performance to specific applications, EWPH is a promising fat replacer in frozen desserts.

##### 3.1.1.3 Plasma proteins

Plasma proteins are derived from the blood plasma of animals, most commonly from bovine (cattle) or porcine (pig) sources (Ofori and Hsieh 2014). These proteins are primarily composed of albumins, globulins, and fibrinogen (Qian 2009), which are known for their excellent emulsifying and foaming properties, making them suitable for fat replacement in frozen desserts. For example, Csurka et al. (2023) investigated the impact of incorporating various animal protein sources with high biological value, including plasma proteins, on the technofunctional properties of ice creams. This study indicated that such enrichment significantly influenced the rheological attributes and texture of ice cream without altering its overall rheological behavior, suggesting the potential of plasma proteins as fat replacers in frozen desserts. Additionally, it is important to note that the effectiveness of plasma proteins as fat replacers in frozen desserts may vary depending on factors, such as protein concentration, processing conditions, and interactions with other ingredients. In summary, plasma proteins show strong potential as fat replacers in frozen desserts because of their emulsifying and foaming abilities, with their effectiveness depending on the formulation and processing factors.

#### 3.1.2 Plant Proteins

Among plant proteins, soy, corn zein, and pea proteins are increasingly being explored as fat replacers in frozen desserts because of their functional properties (Table 2). Incorporating plant proteins into frozen desserts offers several advantages, primarily due to their nutritional and environmental benefits (Fu et al. 2023; Hasan et al. 2024). Nutritionally, these proteins are essential for human growth and daily activities, providing a healthy alternative to animal proteins (Fu et al. 2023). They enhance the nutritional value of foods by supplying essential amino acids and improving their functional and structural properties, which are crucial for developing nutritious food products (Qin et al. 2022). Environmentally, plant protein production is more sustainable than animal protein production because it requires fewer resources and aligns with low‐carbon and sustainable development goals (Fu et al. 2023). However, some plant proteins have limitations, such as off‐flavors, allergenic potential, and antinutritional factors (Fu et al. 2023; Tang et al. 2024), which can negatively affect the overall sensory experience of frozen desserts and reduce the bioavailability of other nutrients. Therefore, further research is needed to improve their suitability as fat replacers in frozen desserts.

##### 3.1.2.1 Soy Proteins

Soy proteins derived from soybeans, particularly soy protein isolates (SPIs), are widely used as plant‐based protein sources (Singh et al. 2008). SPI is prepared by extracting and purifying proteins from defatted soy flour, resulting in more than 90% protein content on a dry basis (Zheng et al. 2022). This process often includes steps such as solubilization, precipitation, and drying, which can be further modified through various physical, chemical, and biological techniques to enhance its functional properties, such as solubility and emulsification (Petruccelli and Anon 1994). In frozen desserts, SPI serves as a protein‐based fat replacer that is crucial for preserving the textural and sensory properties typically provided by fat, such as creaminess, mouthfeel, and flavor. For example, X. Liu et al. (2024) investigated the effects of SPI particle size and mixture viscosity on the structural and sensory properties of fat‐free ice cream. They observed that medium‐sized SPI particles (approximately 4 µm) had improved textural and sensory attributes, making them potential fat replacers in frozen desserts. However, SPI incorporation can alter the flavor profile by introducing green/grassy and doughy/fatty notes, which may affect consumer acceptance (Friedeck et al. 2003). Therefore, the successful application of SPI in frozen desserts requires careful formulation to balance its functional benefits and potential sensory drawbacks, ensuring a product that meets both nutritional and sensory requirements.

##### 3.1.2.2 Zein

Zein, the major storage protein in corn, accounts for approximately 80% of the total protein content of corn (Corradini et al. 2014). It is characterized by its hydrophobicity and unique solubility, which is attributed to its high content of hydrophobic amino acids, such as leucine, proline, and alanine (Giteru et al. 2021). Furthermore, zein can undergo enzymatic hydrolysis using alcalase, which improves its interfacial properties and emulsifying activity (J.‐L. He et al. 2023). Specifically, alcalase‐hydrolyzed zein exhibited improved emulsifying properties, with an emulsifying activity of 66.76 m^2^/g and stability of 78.51 min (Zhang et al. 2023), making it a promising fat replacer in frozen desserts. Zhang et al. (2023) used alcalase‐hydrolyzed zein as a fat replacer in ice cream with 10% fat replacement to improve emulsifying properties and reduce lipid oxidation, resulting in a product with rheological properties and flavor similar to full‐fat ice cream. However, the enzymatic modification of zein should be carefully controlled to maintain desirable physicochemical properties without compromising the texture or flavor of the frozen desserts. Overall, although the use of alcalase‐hydrolyzed zein requires careful control of the enzymatic modification process, its unique functional properties and enhanced emulsifying capabilities make it a promising fat replacer in frozen desserts.

##### 3.1.2.3 Pea Protein

Pea protein, derived from *Pisum sativum* L., is a plant‐based protein source with high nutritional value and low allergenicity (Lam et al. 2018). The primary components of pea protein are globulin and albumin, which are soluble in salt solutions and water, respectively, and contribute to its functional properties, such as hydration and emulsification. Pea protein isolate (PPI) is prepared by extracting these proteins, typically involving processes such as solubilization, precipitation, and drying to concentrate the protein content while removing nonprotein components (Lu et al. 2020). PPI exhibits various functional properties, including solubility, WHC, oil holding capacity (OHC), and emulsifying and foaming abilities (Ge et al. 2020), all of which are crucial for its application in the production of frozen desserts. For example, Guler‐Akin et al. (2021) found that replacing milk powder with pea protein isolate in ice cream at low ratios (6% and 12%) improved the physical and textural properties, whereas high ratios (25% and 100%) negatively affected the sensory attributes. Despite these challenges, the nutritional benefits and functional properties of PPI make it a valuable ingredient for developing healthy frozen desserts.

### 3.2 Carbohydrate‐Based Fat Replacers

**FIGURE 3:**
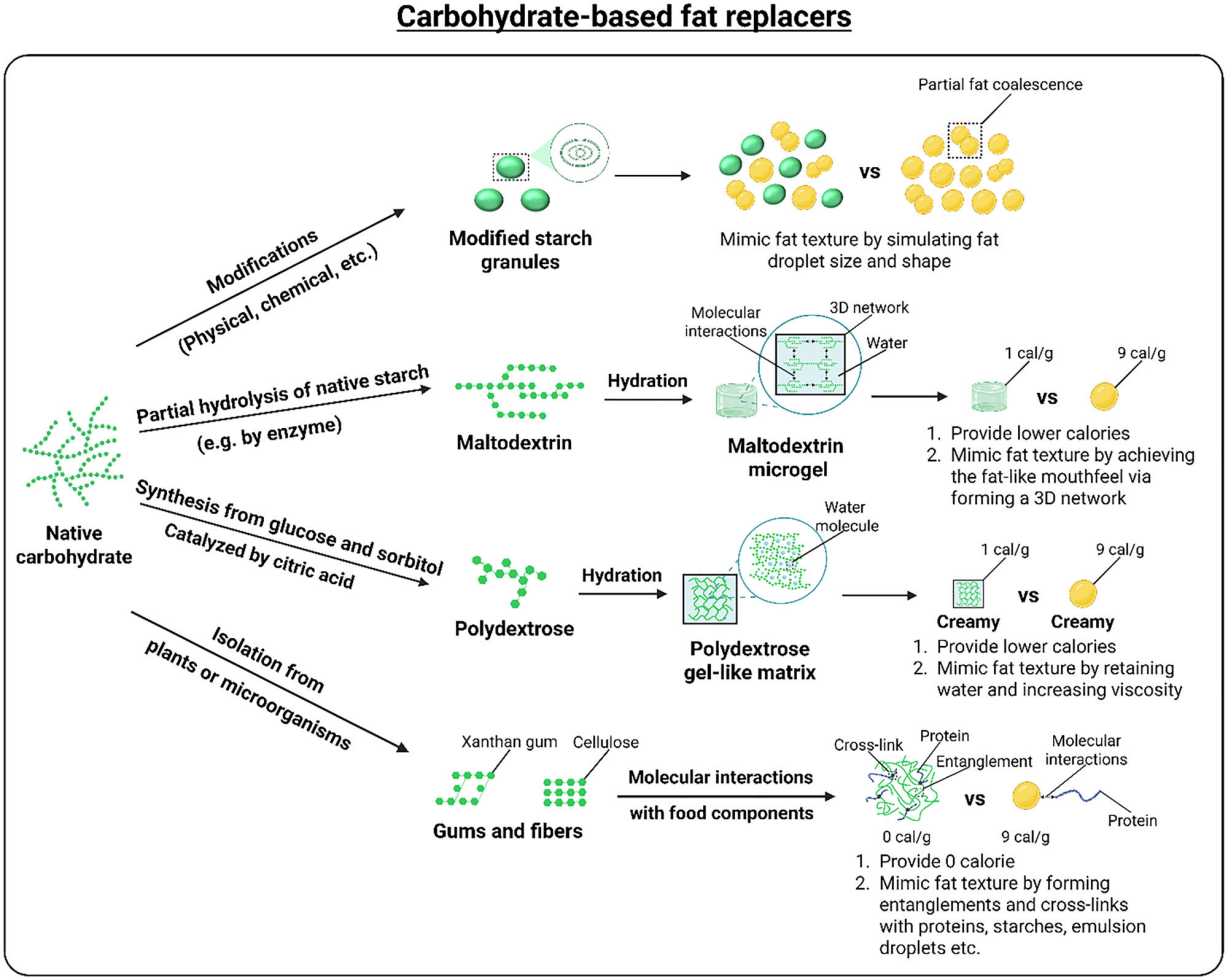
Schematic showing the mechanism of carbohydrate‐based fat replacers in frozen desserts compared to their full‐fat counterparts. *Source*: Created with BioRender.com.

Carbohydrate‐based fat replacers include starch, maltodextrin (MD), polydextrose (PD), gums, and fibers (Peng and Yao 2017). Figure 3 illustrates the four key mechanisms by which carbohydrate‐based fat replacers function in frozen desserts to mimic the texture and mouthfeel of their full‐fat counterparts while reducing their calorie content. Modified starch granules, derived through modifications (physical, chemical, etc.) of native starch, achieve a fat‐like texture to simulate the size and shape of fat droplets (Peng and Yao 2017). Similarly, MD microgels, formed via hydration of MD (produced by partial enzymatic hydrolysis of native starch), create a fat‐like mouthfeel by forming a three‐dimensional (3D) network that traps water (Loret 2004), thereby reducing calorie content to 1 cal/g compared to 9 cal/g of fat (Méndez‐Velasco and Goff 2012; Azari‐Anpar et al. 2017; Chen et al. 2020; Cruz et al. 2022). PD gel‐like matrices, synthesized from glucose and sorbitol catalyzed by citric acid (CA), mimic fat texture by retaining water and increasing viscosity, providing 1 cal/g (da Silva et al. 2020; Huang et al. 2020). Additionally, gums and fibers (e.g., xanthan gum [XG], cellulose) isolated from plants or microorganisms achieve a fat‐like texture through molecular interactions with food components, forming entanglements and cross‐links with proteins, starches, and emulsion droplets, while contributing zero calories (Peng and Yao 2017). These mechanisms collectively enable carbohydrate‐based fat replacers to reduce calorie intake while maintaining the desirable sensory attributes of full‐fat frozen desserts (Table 3).

**TABLE 3:** Applications of carbohydrate‐based fat replacers in frozen desserts.

Types	Forms	Applications	Suggested replacement ratio (%)	Functions	References
Starches	Small‐granule starches (SGSs) from wheat and amaranth	Vanilla ice cream	50 (with 2% SGSs from wheat and amaranth)	Provide sensory acceptability similar to the full‐fat control	(Malinski et al. 2003)
	High‐pressure homogenization and ultrasonication (HPH‐US)‐treated corn starch nanocrystals (CSNCs)	Milk ice cream	20	Decrease melting rate and hardness, achieve sensory score similar to the full‐fat control	(Lin et al. 2023)
	Acetylated pea starch (APS)	Vanilla ice cream	50 (with 5.07% APS)	Improve texture and sensory qualities	(Aime et al. 2001)
	Octenyl succinyl anhydride (OSA)‐esterified pearl millet starch (PMS)	Milk ice cream	25, 50 (with 1% or 2% OSA‐esterified PMS)	Increase consistency index values and apparent viscosity, enhance structural strength and sensory qualities	(Sharma et al. 2017)
	Citric acid (CA)‐treated sweet potato starch (SPS)	Vanilla ice cream	≈45, 91 (with 1% CA‐treated SPS)	Achieve high sensory acceptance, hold physicochemical characteristics similar to the full‐fat control	(Surendra Babu et al. 2018)
	Cross‐linked cassava starch (CS)	Milk ice cream	30 (with 10% cross‐linked CS)	Increase water holding capacity (WHC), viscosity, overrun, and hardness, decrease melting rate	(Marimon‐Valverde et al. 2024)
Maltodextrin (MD)	MD with 10 dextrose equivalent (MD‐10)	Vanilla ice cream	≈14 (with 2% MD‐10)	Increase mix viscosity, provide color, texture, and cream flavor similar to the full‐fat control, maintain consumer acceptability	(Roland et al. 1999; Rolon et al. 2017)
Polydextrose (PD)	Litesse^®^	Cherry ice cream	50 (with 6% Litesse^®^)	Increase cream flavor release, achieve sensory and texture properties similar to the full‐fat control	(Chung et al. 2004)
Gums	Locust bean gum (LBG)	Vanilla ice cream	90 (with 0.55% LBG)	Increase viscosity and overrun, decrease hardness, achieve textural and sensory properties similar to the full‐fat control	(Liu et al. 2023)
	Guar gum (GG)	Orange frozen yogurt	≤58 (with ≤0.3% GG)	Increase viscosity, overrun, and melting stability, decrease hardness, enhance sensory qualities	(Milani and Koocheki 2011)
		Vanilla ice cream	75 (with 0.35% GG)	Increase vanilla flavor, achieve similar overrun to the full‐fat control	(Javidi et al. 2016)
	Pectin	Milk ice cream	45 (with 0.72% pectin)	Increase viscosity, overrun, hardness, and smoothness, decrease melting rate	(Zhang et al. 2018)
	Basil seed gum (BSG)	Vanilla ice cream	50 (with 0.45% BSG)	Decrease coarseness, coldness, and melting rate	(Javidi and Razavi 2018)
Fibers	β‐Glucan from oat	Milk ice cream	80 (with 0.5% β‐glucan)	Provide creaminess, suppress ice crystal formation, increase viscosity, overrun, and decrease melting rate	(Buniowska‐Olejnik et al. 2023)
	Inulin	Frozen yogurt	50 (with 5% inulin)	Increase WHC, decrease melting rate and softening rate	(El‐Nagar et al. 2002)
		Milk ice cream	≈14 (with 4% inulin)	Increase firmness, overrun, flavor score, and viscosity, improve air incorporation	(Akalın and Erişir 2008; Samakradhamrongthai et al. 2021)
Fibers	Cereal fiber from oat	Vanilla ice cream	≈33	Increase WHC and firmness, decrease overrun	(Tolve et al. 2024)
	Fruit fiber from orange	Chocolate ice cream	70 (with 0.74% orange fiber)	Increase WHC and oil holding capacity (OHC), maintain color, odor, and texture similar to the full‐fat control	(de Moraes Crizel et al. 2013)
	Cellulose nanofibrils (CNFs)	Milk ice cream	50 (with 0.3% CNFs)	Increase viscosity, improve melting rate, and mouthfeel, decrease friction of ice crystals, cover unfavorable sensation	(Velásquez‐Cock et al. 2019)
	Grapefruit peel nanofibrillated cellulose (GNFC)	Milk ice cream	17.9 (with 0.4% GNFC)	Increase hardness, chewiness, and WHC, decrease melting rate, inhibit protein and fat digestion, form stable suspension	(Yu et al. 2021)
	Hydroxypropyl methylcellulose (HPMC)	Vanilla probiotic ice cream	≈46 (with 0.3% HPMC)	Improve creamy sensation, melting qualities and flavor	(Turgut and Cakmakci 2009; Soukoulis et al. 2010)

#### 3.2.1 Starches

Starches, widely distributed in nature as common components of higher plants, serve as primary carbohydrate‐based fat replacers (Table 3). Based on their particle size, starches can be divided into four categories: large (>25 µm), medium (10–25 µm), small (5–10 µm), and very small (<5 µm) granular starches (Lindeboom et al. 2004). Small and very small granule starches are collectively referred to as small‐granule starches (SGSs). Studies have shown that the minimum particle size of SGSs can reach 2 µm or even smaller, which is similar to the size of fat particles, imparting them with a fat‐like taste (Ma et al. 2006; Javidi et al. 2019). The interaction of SGSs with saliva and other components (such as proteins and lipids present in the food matrix) during oral processing is complex and involves several mechanisms. The high specific surface area of SGSs enhances their ability to form oleogels through capillary forces, which can transition from a fluid‐like to a gel‐like state, thereby influencing the lubrication performance and sensory perception of food products (Han et al. 2024). Malinski et al. (2003) discovered that SGSs (2.0–10.0 µm) from wheat and amaranth could replace up to 50% of the fat in frozen desserts without significantly affecting sensory attributes such as smoothness, creaminess, and preference. However, native starches have limitations, including poor stability under high shear and temperature conditions (Abbas et al. 2010). To overcome these shortcomings, various starch modifications have been developed, including physical, chemical, enzymatic, genetic, and dual modifications (R. He et al. 2023; Sinhmar et al. 2023). In frozen desserts, modified starches used as fat replacers are primarily obtained through physical or chemical modifications (Table 3).

Physical modifications, such as hydrothermal treatment, microwaves, pregelatinization, ball milling, ultrasonication (US), and high hydrostatic pressure (HHP), are ecofriendly techniques that alter the crystalline properties of starch granules, thereby enhancing their functional characteristics for industrial applications (R. He et al. 2023; Sudheesh et al. 2024). These modifications can increase the solubility, swelling power, and gelatinization (N. Wang et al. 2023), making starches suitable as fat replacers in frozen desserts. Lin et al. (2023) investigated the use of high‐pressure homogenization and US (HPH‐US)‐treated corn starch nanocrystals (CSNCs) as fat replacers in ice cream. The study found that replacing 20%–100% of the fat with HPH‐US‐treated CSNCs increased ice cream viscosity and hardness while decreasing the melting rate, with 20% replacement being the most preferred by sensory evaluators.

In addition to physical modifications, chemical modifications are essential for native starches to act as effective fat replacers in frozen desserts. Primary chemical modifications include oxidation, acetylation, etherification, esterification, acid hydrolysis, and cross‐linking, which introduce functional groups into starch molecules and enhance their physicochemical properties (Gałkowska et al. 2023; R. He et al. 2023). For example, Aime et al. (2001) investigated the impact of using modified pea starch as a fat replacer in ice cream with varying fat levels and discovered that light (5%) ice cream maintained sensory attributes comparable to regular fat (10%) ice cream. However, the low‐fat (2.5%) and fat‐free (0.4%) versions exhibited lower viscosity, smoothness, and mouth coating properties. Sharma et al. (2017) evaluated octenyl succinyl anhydride (OSA)‐esterified pearl millet starch (PMS) as a fat replacer in ice cream using 1% and 2% levels in mixes with 7.5% and 5% fat, respectively. The results showed that OSA‐esterified PMS maintained sensory acceptability and rheological characteristics comparable to those of other fat replacers, including inulin and WPC‐70, while also offering potential health benefits owing to its resistant starch (RS) content. Surendra Babu et al. (2018) investigated the use of CA‐treated sweet potato starch (SPS) as a fat replacer in ice cream and found that 1% CA‐treated SPS improved the texture of medium‐fat (6%) and low‐fat (1%) ice creams, making them comparable to full‐fat (11%) ice cream in sensory evaluations after 60 days of storage.

In summary, SGSs are effective fat replacers in frozen desserts because of their small particle size and ability to enhance sensory attributes. However, native starches have stability issues under high shear and temperature conditions. Physical and chemical modifications, such as hydrothermal treatment and acetylation, improve their functional properties, resulting in better viscosity, hardness, and sensory acceptability of reduced‐fat‐frozen desserts.

#### 3.2.2 Maltodextrins

MDs are low‐sweet saccharide polymers derived from starch hydrolysis, consisting of D‐glucose units linked primarily by α‐1,4 bonds with some α‐1,6 branching (Hofman et al. 2016). The structures and properties of MDs vary depending on the source, with rice MDs containing more low‐molecular‐weight (LMW) saccharides and potato MDs containing high‐molecular‐weight (HMW) components (Wang and Wang 2000). As fat replacers in frozen desserts, MDs exhibit strong WHC and can form thermoreversible gels with water, thereby replicating the fat mouthfeel (Chen et al. 2020). Roland et al. (1999) studied the effects of MD with 10 dextrose equivalent (MD‐10) as a fat replacer in ice cream and used it to formulate fat‐free ice cream with 0.5% milk fat or less. The results showed that MD‐10 improved the appearance and texture of ice cream compared to 0.1% fat ice cream and was closest to the 10% fat ice cream in terms of cream flavor and textural characteristics. Similarly, Rolon et al. (2017) investigated the effects of replacing fat with MD in vanilla ice cream and observed that substituting 6%–14% fat with 0%–8% MD altered the physical properties but did not significantly affect the consumer acceptability of both fresh and stored ice creams. However, the hygroscopic nature of MDs can lead to moisture absorption (Chronakis 1998), which may affect the stability and shelf life of the frozen desserts. In conclusion, MDs are effective fat replacers in frozen desserts because of their excellent water‐holding capacity and gel‐forming ability, although their performance depends on their molecular weight, botanical origin, and hygroscopic nature.

#### 3.2.3 Polydextrose

PD is a low‐calorie, nondigestible polysaccharide synthesized from glucose, sorbitol, and CA (Craig 2000). Its high water solubility and stability over a broad pH and temperature range make it suitable for various food applications (Veena et al. 2016). In frozen desserts, Litesse^®^ (an improved PD) and Simplesse^®^ were evaluated for their effects on flavor release in reduced‐ and full‐fat ice creams, and it was found that Litesse^®^ showed sensory properties closer to those of full‐fat ice cream than Simplesse^®^ in the full‐fat group (Chung et al. 2004). Roland et al. (1999) found that incorporating Litesse^®^ as a fat replacer in ice cream resulted in similar stickiness (ease of scooping) compared to the 10% fat sample; however, this formulation exhibited a faster melting rate than the full‐fat control and was perceived to have a stronger corn syrup flavor and aftertaste than the 0.1% fat control, as well as a more intense bitterness compared to all other ice cream samples. These findings indicate that while Litesse^®^ may have some positive effects on certain textural aspects of frozen desserts, such as stickiness, it also presents several drawbacks in terms of the flavor and melting characteristics of the products. These drawbacks may negatively affect the consumer acceptance of Litesse^®^‐formulated frozen desserts. Therefore, further research is needed to optimize the use of Litesse^®^ and to explore alternative fat replacers.

#### 3.2.4 Gums

Gums are a class of polysaccharides widely used as fat replacers in frozen desserts owing to their thickening and stabilizing properties (Table 3). These polysaccharides can be classified into flexible polysaccharides, such as locust bean gum (LBG) and guar gum (GG), and rigid polysaccharides, such as XG, ι‐carrageenan (ι‐C), and alginate (Picout et al. 2001, 2002; Thrimawithana et al. 2010). Flexible polysaccharides exhibit lower shear‐thinning behavior and more liquid‐like viscoelastic properties than rigid polysaccharides. In ice cream, flexible polysaccharides (LBG and GG) led to higher overrun, lower hardness, and improved softness and creaminess, whereas rigid polysaccharides (XG and ι‐C) resulted in higher coldness and grittiness (Liu et al. 2023). However, rigid polysaccharides can lead to gelation of the serum phase, making it more difficult to scoop ice cream. Therefore, the selection of appropriate gums as fat replacers in frozen desserts should consider their structural characteristics and functional effects to achieve the desired balance of texture, stability, and sensory attributes in the final product.

#### 3.2.5 Fibers

Fibers, including β‐glucan, inulin, cereal and fruit fibers, and cellulose derivatives, are increasingly being employed as fat replacers in frozen desserts because of their unique properties that effectively mimic the texture and mouthfeel of fat (Table 3). By increasing viscosity and stabilizing the emulsion, these fibers help maintain the creamy texture and mouthfeel typically provided by fat (Peng and Yao 2017). Additionally, they can improve the aeration and overrun of ice cream, resulting in lighter and more palatable products. The interaction of these fibers with other ingredients, such as proteins and sugars, further enhances their functional properties (Brennan and Tudorica 2008), allowing them to effectively replace fat while preserving the quality and sensory attributes of the frozen desserts.

##### 3.2.5.1 β‐Glucan

β‐Glucan is a nonstarchy polysaccharide composed of D‐glucose units linked by β‐(1,3) and β‐(1,4) glycosidic bonds (Hu et al. 2015; Lante et al. 2023). It alters the functional characteristics of food products, including their viscosity, rheology, and texture (Kaur et al. 2020). Buniowska‐Olejnik et al. (2023) discovered that oat β‐glucan effectively reduced ice crystal growth and enhanced the quality attributes of low‐fat ice cream, making it a promising fat replacer in frozen desserts. This study demonstrated that β‐glucan increased the overrun, resistance to melting, and sensory appeal of ice cream, bringing it closer to the quality of full‐fat ice cream. The functionality of β‐glucan in frozen desserts is primarily attributed to its ability to form viscous solutions upon solubilization, facilitated by the presence of β‐1,3 linkages that break up a uniform structure (Hu et al. 2015). It also offers health benefits, including cholesterol reduction and blood glucose regulation, which have been recognized by regulatory authorities (Lante et al. 2023). However, a key challenge in using β‐glucan as a fat replacer is its degradation via oxidation during processing (Mejía et al. 2020). Therefore, careful processing is essential when β‐glucan is incorporated into frozen desserts as a fat replacer to maintain their functional properties.

##### 3.2.5.2 Inulin

Inulin is a fructan‐type polysaccharide composed of β‐D‐fructosyl residues linked by (2→1) bonds, often with an α‐D‐glucose end group (Mensink et al. 2015). It is widely distributed in nature as a plant storage carbohydrate, with chicory roots being the richest source (Shoaib et al. 2016). The physicochemical properties of inulin, including its solubility, viscosity, and gelling ability, make it a suitable fat replacer in frozen desserts. El‐Nagar et al. (2002) found that the addition of inulin to yog‐ice cream not only increased its viscosity and hardness but also improved its meltdown properties and sensory attributes. Akalın and Erişir (2008) investigated the effects of inulin and oligofructose on the rheological properties and probiotic survival of low‐fat ice cream. They discovered that inulin addition significantly improved the firmness, melting properties, and survival of ice cream compared to oligofructose and control samples did. Samakradhamrongthai et al. (2021) optimized a reduced‐fat ice cream formulation using inulin as the fat replacer, achieving a 2.30% fat reduction and acceptable sensory qualities. Overall, inulin offers promising functional properties for fat replacement in frozen desserts.

##### 3.2.5.3 Cereal and Fruit Fibers

Cereal fibers from sources such as oats, wheat, and corn bran (Soukoulis et al. 2009; Rose et al. 2010) and fruit fibers from sources such as apples, oranges, and pitaya (Soukoulis et al. 2009; de Moraes Crizel et al. 2013; Utpott et al. 2020) are rich in dietary fiber and bioactive compounds. Tolve et al. (2024) studied the effects of inulin, acacia, oat, and apple fibers on the physical, thermal, and sensory properties of low‐fat ice cream and found that inulin and acacia fibers yielded characteristics similar to those of the full‐fat ice cream. Additionally, de Moraes Crizel et al. (2013) showed that orange byproduct fibers, with their high WHC and OHC and rich phenolic and carotenoid content, can reduce the fat content in ice cream by 70% without significantly affecting its color, odor, or texture. These studies suggest the potential use of cereal and fruit fibers as fat replacers in reduced‐fat frozen desserts production.

##### 3.2.5.4 Cellulose Derivatives

Cellulose derivatives are widely used as fat replacers in frozen desserts (Table 3). These derivatives can be categorized into three main types: (i) chemically and mechanically treated cellulose, such as microcrystalline cellulose (MCC), nanocrystalline cellulose (NCC), and cellulose nanofibrils (CNFs) (Velásquez‐Cock et al. 2019); (ii) chemically modified cellulose, such as methylcellulose (MC), carboxymethyl cellulose (CMC), and hydroxypropyl methylcellulose (HPMC) (He et al. 2021); and (iii) biologically modified cellulose, such as bacterial cellulose (BC) (Okiyama et al. 1993). For example, Velásquez‐Cock et al. (2019) explored the impact of incorporating CNFs at 5% and 10% fat concentrations in ice cream and found that CNFs enhanced the viscosity of the mixture and improved the sensory properties of low‐fat ice cream. Similarly, Yu et al. (2021) investigated the use of grapefruit peel nanofibrillated cellulose (GNFC) as a fat replacer in ice cream and observed that it significantly improved the textural and sensory properties of ice cream. Their study showed that ice cream with 0.4% GNFC exhibited desirable three‐dimensional network structures and received the highest sensory evaluation score. GNFC also reduced the gross energy (GE) of ice cream by up to 17.90%, indicating a lower calorie content of the ice cream. Furthermore, Soukoulis et al. (2010) observed that HPMC at 0.3 g/100 g improved the creamy sensation and textural qualities of probiotic ice cream, enhancing flavor release without affecting overall acceptability. Together, these studies highlight the functions of cellulose derivatives, making them valuable thickeners, emulsion stabilizers, and functional ingredients in the preparation of frozen desserts.

### 3.3 Lipid‐Based Fat Replacers

**FIGURE 4:**
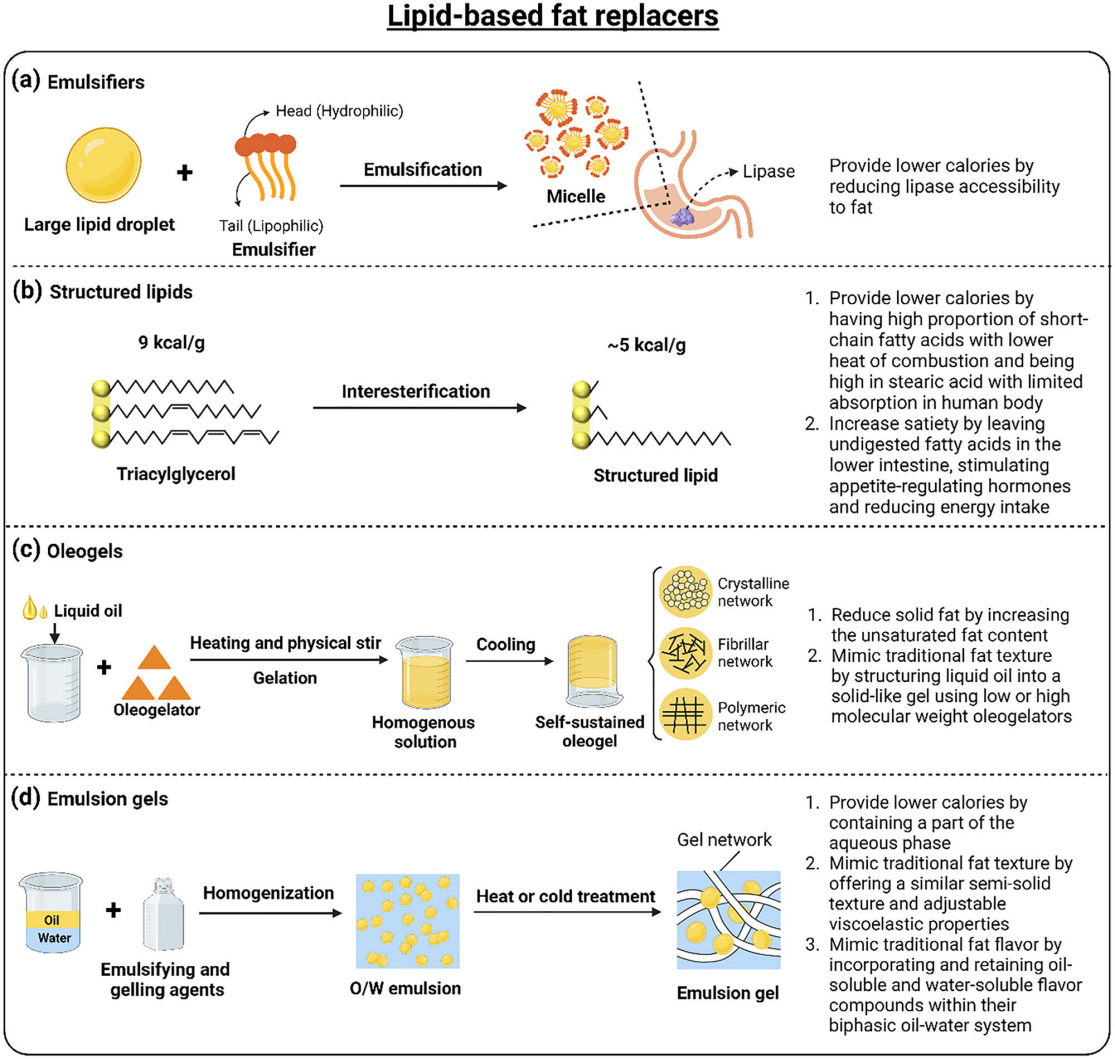
Schematics showing the mechanism of lipid‐based fat replacers in frozen desserts: (a) emulsifiers; (b) structured lipids; (c) oleogels; (d) emulsion gels. *Source*: Created with BioRender.com.

Lipid‐based fat replacers include emulsifiers, structured lipids (SLs), oleogels, and emulsion gels (Guo et al. 2023; Yazar and Rosell 2023). Lipid‐based fat replacers in frozen desserts function through four mechanisms to reduce calorie content while mimicking the sensory attributes of traditional fats (Figure 4). Emulsifiers (Figure 4a) reduce calorie intake by physically disrupting fat droplets into smaller particles, forming micelles that limit lipase accessibility to triglycerides, thereby inhibiting fat digestion without altering the creamy texture of frozen desserts (Reis et al. 2009; McClements and Jafari 2018). SLs (Figure 4b) achieve calorie reduction by incorporating short‐chain fatty acids with lower heat of combustion and stearic acid with limited absorption in the human body (Sørensen et al. 2008). Undigested fatty acids in the lower intestine further enhance satiety by stimulating appetite‐regulating hormones (Osborn and Akoh 2002). Oleogels (Figure 4c) replace solid fats by structuring liquid oils into self‐sustained gels through crystalline, fibrillar, or polymeric networks formed via heating and physical stirring, effectively reducing the saturated fat content while maintaining the traditional fat texture (Davidovich‐Pinhas et al. 2016; L. Li et al. 2022; Banaś et al. 2023; Guo et al. 2023; Hu et al. 2023; Z. Wang et al. 2023). Emulsion gels (Figure 4d) simultaneously address calorie reduction and texture mimicry by forming viscoelastic networks that retain both oil‐soluble and water‐soluble flavor compounds, thereby preserving the authentic flavor profile of full‐fat products (Ren et al. 2022; Guo et al. 2023; Hu et al. 2023). In summary, these lipid‐based fat replacers address the challenges of fat reduction in frozen desserts by balancing calorie reduction, texture mimicry, and flavor retention (Table 4).

**TABLE 4:** Applications of lipid‐based fat replacers in frozen desserts.

Types	Sources	Forms	Applications	Suggested replacement ratio (%)	Functions	References
Emulsifiers	Monoglycerides (MGs)	Glycerol monooleate (GMO)	Vanilla ice cream	60 (with 0.3% GMO)	Increase overrun, decrease melting rate, provide sensory properties similar to the full‐fat control	(Zeng et al. 2012)
Structured lipids (SLs)	Diacylglycerols (DAG)	Camellia oil‐based DAG (CD) solid fractions	Vanilla ice cream	≈49 (with 5.11% fractionated CD)	Increase overrun and hardness, maintain desirable melting property at human mouth temperature, improve smoothness through increased level of β’‐form crystals	(Yang et al. 2022)
Oleogels	Low molecular weight oleogelators (LMWOs)	Beeswax (BW)	Milk ice cream	100 (with medium chain triglyceride (MCT) and 7% BW)	Promote fat partial coalescence, increase overrun and structural strength, decrease melting rate, maintain shape, provide smooth appearance	(N. Liu et al. 2024)
		Rice bran wax (RBW)	Milk ice cream	100 (with high oleic sunflower oil (HOSO) and 10% RBW)	Structure edible oil, enhance appearance, increase overrun and fat partial coalescence, decrease melting rate	(Zulim Botega et al. 2013a, 2013b)
		Candelilla wax (CDW)	Plant‐based milk ice cream	100 (with hemp seed oil (HO) and 9% CDW)	Increase hardness and OHC, decrease adhesiveness, achieve high sensory acceptability	(Ropciuc et al. 2024)
Oleogels	Low molecular weight oleogelators (LMWOs)	Carnauba wax (CBW)	Vanilla ice cream	50 (with soybean oil (SBO) and 6% CBW)	Reduce melting rate, achieve appearance, creaminess, and texture similar to the full‐fat control	(Airoldi et al. 2022)
		Phytosterol and γ‐oryzanol (PS‐GO)	Milk ice cream	100 (with sunflower oil (SO) and 12% PS‐GO)	Increase overrun and apparent viscosity, delay melting, enhance shape retention	(Moriano and Alamprese 2017)
				100 (with MCT and 10% PS‐GO)	Promote fat partial coalescence, delay melting, reduce crystal size, increase overrun and firmness	(N. Liu et al. 2024)
	High molecular weight oleogelators (HMWOs)	Whey protein (WP)	Vanilla ice cream	100 (with high oleic palm oil (HOPO) and 4% WP)	Increase viscosity, cohesiveness, chewiness, and gumminess, reduce adhesiveness, exhibit similar hardness and springiness similar to the full‐fat control	(Silva‐Avellaneda et al. 2021)
		Hydrolyzed collagen	Milk ice cream	100 (with PS and 5% hydrolyzed collagen)	Increase overrun and protein content, decrease melting rate, achieve overall qualities similar to the full‐fat control	(Santos and Lannes 2022)
Oleogels	High molecular weight oleogelators (HMWOs)	Ethylcellulose (EC)	Milk ice cream	100 (with HOSO and 10% EC)	Prevent large crystal growth, achieve small and evenly distributed fat globules similar to the full‐fat control	(Munk et al. 2018)
Emulsion gels	Protein‐based emulsion gels	Pickering emulsion stabilized by pea protein microgel (PPM) prepared at pH 7.0	Milk ice cream	60	Decrease oil droplet size, increase structural stability, achieve melting properties, overrun, and sensory properties comparable to the full‐fat control	(Qin et al. 2024)
	Polysaccharide‐based emulsion gels	Walnut oil emulsion gel based on blueberry pectin and CaCl_2_	Milk ice cream	100	Exhibit melting rate similar to the full‐fat control, increase viscosity and density of the structure, achieve desirable texture and taste	(Huang et al. 2024)
	Mixed emulsion gels	Double emulsion gel emulsified by polyglycerol poliricinoleate (PGPR)‐lecithin blend and stabilized by guar gum‐gum tragacanth (GGGT) blend	Vanilla ice cream	≈77	Decrease melting rate, increase overrun and overall acceptability	(Tekin et al. 2017)
Emulsion gels	Mixed emulsion gels	Low oil Pickering emulsion gel stabilized by bacterial cellulose nanofibers (BCNs) and SPI	Milk ice cream	90 (with 5% oil phase fraction Pickering emulsion gel)	Achieve appearance, smell, and morphology similar to the full‐fat control, decrease melting rate, hold ability to recover deformation after damage	(Gao et al. 2023)

#### 3.3.1 Emulsifiers

Emulsifiers, such as monoglycerides (MGs), mono‐ and diglycerides (MDG), and polyglycerol fatty acid esters (PGFEs), play a crucial role in the formulation of low‐fat foods as lipid‐based fat replacers (Oreopoulou 2006; Yazar and Rosell 2023). Among these, MGs have been used as fat replacers in frozen desserts (Table 4). Owing to their unique self‐assembly properties in water, oil, and multiphase environments, MGs are valuable structuring molecules that can produce a wide range of fat alternatives and effectively mimic the textural properties of traditional fats (Melchior et al. 2024). MGs can be categorized into saturated types, such as glycerol monostearate (GMS) and glycerol monopalmitate (GMP), and unsaturated types, such as glycerol monooleate (GMO) (Zeng et al. 2012; Wang and Marangoni 2016). Zeng et al. (2012), synthesized GMO via the glycerolysis of camellia oil using lipase Novozym 435 and incorporated it into low‐fat ice cream at a concentration of 0.3%. The study found that GMO effectively bridged the sensory gap between low‐fat (4%) and full‐fat (10%) ice creams, enhancing both the overrun and melting resistance. These results suggest that GMO has the potential to improve the quality of reduced‐fat ice cream. However, further research is needed to explore the potential of other emulsifiers as fat replacers in frozen desserts.

#### 3.3.2 Structured Lipids

SLs are functional lipids designed to alter the fatty acid composition and distribution on the glycerol backbone through chemical or enzymatic processes (Osborn and Akoh 2002; Kim and Akoh 2015). Diacylglycerols (DAGs) are a type of SLs that can form stable emulsions and improve the texture of frozen desserts while also offering reduced caloric content. Yang et al. (2022) studied the use of fractionated camellia oil‐based diacylglycerol (CD) in vanilla ice cream production. This study revealed that fractionation increased the solid fat content (SFC) of CD, thereby improving its suitability for use in ice cream. The ice cream made with CD solid fractions exhibited better overrun, hardness, and smoother texture owing to the higher levels of β’‐form crystals compared to those made with camellia oil or unfractionated CD. However, DAG has some drawbacks, including lower oxidative and thermal stability than conventional oils (W. J. Lee et al. 2020). Furthermore, safety concerns have been raised regarding DAG, particularly regarding the potential formation of glycidol fatty acid esters (GEs), which may be carcinogenic (Y.‐Y. Lee et al. 2020). In conclusion, although SLs offer promising functional benefits in frozen dessert applications, their limitations in terms of stability and potential health risks must be carefully evaluated to ensure their safe consumption.

#### 3.3.3 Oleogels

Oleogels are novel fat replacers that replicate the properties of solid fats using liquid oils, presenting a healthier alternative to trans and saturated fats in frozen desserts (Table 4). They can be prepared using direct (hot or cold) or indirect methods, such as emulsion‐templated, foam‐templated, hydrogel‐templated, and solvent exchange methods (Pinto et al. 2024). Oleogels can be classified into low molecular weight oleogelators (LMWOs) and high molecular weight oleogelators (HMWOs) based on the molecular weight of their oleogelators (Kavya et al. 2024). LMWOs include mono‐ and multicomponent oleogels, whereas HMWOs include proteins and polysaccharides (L. Li et al. 2022). The oleogelation mechanism and composition significantly influenced the physicochemical properties and functionality of the oleogels (L. Li et al. 2022), allowing for tailored applications in frozen desserts.

##### 3.3.3.1 Oleogels Prepared with LMWOs

Oleogels prepared using LMWOs are promising alternatives to the solid fats used in frozen desserts (Table 4) and can be categorized into mono‐ and multicomponent systems, as previously mentioned. Monocomponent oleogels typically utilize a single oleogelator, such as wax, to form a gel network that encapsulates liquid oils, providing a structure that mimics the texture and rheological properties of solid fats (Z. Wang et al. 2023). For example, Ropciuc et al. (2024) developed a plant‐based ice cream using candelilla wax (CDW) oleogels as fat replacers and found that increasing the wax percentage boosted titratable acidity (TA), with the 9% wax sample receiving the highest acceptability score. This study also characterized the physicochemical and rheological properties of the oleogels and concluded that CDW oleogels could effectively replace animal fat in ice creams. Additionally, multicomponent oleogels combine different oleogelators to enhance structural and sensory properties. Moriano and Alamprese (2017) investigated the use of sunflower oil (SO) oleogels prepared with phytosterols and γ‐oryzanol as fat replacers in artisanal ice creams. They reported that oleogels with higher gelator concentrations can achieve quality characteristics comparable to those of traditional formulations while significantly reducing the saturated fat content. Collectively, oleogels prepared with LMWOs can replace saturated and trans fats in frozen desserts while maintaining desirable textural and sensory properties.

##### 3.3.3.2 Oleogels Prepared with HMWOs

In addition to oleogels structured by LMWOs, those prepared with HMWOs, such as proteins and polysaccharides, are potential fat replacers in frozen desserts (Table 4). Proteins were used to create oleogels with desirable structural characteristics, including fibril formation (K. Wang et al. 2024) and capillary networks (G.‐S. Wang et al. 2024), which enhance their rheological properties and stability. Silva‐Avellaneda et al. (2021) investigated the formation of oleogels using whey protein and various oils and optimized the processing conditions to achieve stable emulsions with low ζ‐potential and average droplet size (ADS) for an ice cream base. The study concluded that oleogels derived from microfluidized whey protein and high oleic palm oil (HOPO) can effectively replace cream in ice cream production, enhancing texture and stability while reducing saturated fat content. Additionally, polysaccharides contribute to the robustness and moduli values of the gel network, thereby improving the textural and rheological behaviors of oleogels (Hashemi et al. 2024). Munk et al. (2018) used oleogels prepared with ethylcellulose (EC) as fat replacers in ice cream made with high oleic sunflower oil (HOSO), aiming to improve the structure by inhibiting droplet coalescence and creating a colloidal fat network. They found that HOSO oleogels effectively inhibited oil droplet coalescence, resulting in smaller droplets and an improved ice cream structure compared to unstructured oils. In summary, oleogels prepared with HMWOs can also mimic the textural and sensory attributes of solid fats while reducing the fat content in frozen desserts.

#### 3.3.4 Emulsion Gels

Emulsion gels are semisolid materials with 3D network structures formed by gelling emulsions using various methods. They are prepared by first creating an emulsion and then inducing gelation using physical, chemical, enzymatic, or combined methods (Lin et al. 2020; Zhi et al. 2023). Physical induction methods include heat‐, salt‐, and acid‐induced gelation, whereas chemical methods involve cross‐linking agents, and enzymatic methods use enzymes (Zhi et al. 2023). Combined methods, such as salt‐ and enzyme‐induced gelation, have also been employed (Yan et al. 2021). The preparation technique significantly influences the rheological and textural properties of emulsion gels (Hashemi et al. 2023). Emulsion gels in frozen desserts can act as lipid‐based fat replacers because of their adjustable morphology, microstructure, and functional properties. These characteristics make them ideal for modifying the texture, melting behavior, and sensory qualities of frozen dessert formulations (Table 4).

Furthermore, emulsion gels can be classified into three categories based on their gel matrix composition: protein, polysaccharide, and mixed emulsion gels (Abdullah et al. 2022). Protein‐based emulsion gels, such as those stabilized by pea protein microgels (PPMs) prepared at pH 7.0, can replace up to 60% of the saturated fat in ice cream without compromising structural stability, melting properties, overrun, or sensory attributes (Qin et al. 2024). Additionally, polysaccharide‐based emulsion gels can enhance the natural gel‐forming ability of polysaccharides to create structures with good OHC and thermal stability (Hu et al. 2023), which are essential for preserving the desired mouthfeel and texture of reduced‐fat frozen desserts. For example, Huang et al. (2024) demonstrated that nut oil emulsion gels based on blueberry pectin and CaCl_2_ can effectively replace traditional fats in ice cream, resulting in products with improved physicochemical and sensory properties comparable to those of conventional butter‐based ice creams. As for mixed emulsion gels, they combine proteins and polysaccharides, offering a synergistic approach to enhance the structural and functional properties of gels. For instance, Tekin et al. (2017) evaluated the use of double emulsions emulsified by a polyglycerol poliricinoleate (PGPR)‐lecithin blend and stabilized by GG‐gum tragacanth (GGGT) as fat replacers in ice cream. The results showed that the double emulsion method reduced the fat content to 2.8% without compromising quality, improving overrun, meltdown resistance, and overall sensory acceptability compared to conventional low‐fat ice cream. Similarly, Gao et al. (2023) discovered that low oil Pickering emulsion gels stabilized by BC nanofibers (BCNs) and SPI can effectively replace fat in ice cream, maintaining texture and melting properties similar to those of traditional ice cream while offering improved environmental stability. Despite these advantages, the structural stability of these gels can be influenced by factors such as the concentration of emulsifying agents and interactions between gel components (Jiang et al. 2024), which should be carefully optimized to achieve the desired functionality in frozen desserts. Overall, although emulsion gels are promising fat replacers for frozen desserts, their successful application requires careful consideration of their composition and processing conditions to overcome inherent challenges and achieve the desired product quality.

### 3.4 Complex Fat Replacers

The limitations of using a single protein, carbohydrate (polysaccharide), or lipid‐based fat replacer in frozen desserts are primarily due to their inability to fully replicate the complex sensory and textural properties of fats. Protein‐based fat replacers, such as SPI, can enhance viscosity and foaming ability; however, their effectiveness is highly dependent on particle size and dispersibility, which can affect the texture and melting resistance of ice cream (X. Liu et al. 2024). Polysaccharides, while increasing viscosity, often fail to mimic the smoothness and mouthfeel of fat owing to their structural rigidity, which can lead to undesirable textural properties, such as increased hardness and reduced scoopability in ice cream (Liu et al. 2023). Although lipid‐based fat replacers can impart functional properties to fats, they often do not match the sensory attributes of natural fats and may have metabolic implications (Shaheen et al. 2023). These limitations have led to the development of complex fat replacers that combine multiple components to better mimic the properties of fat in frozen desserts (Table 5). These replacers can be categorized as binary or ternary complexes (Vélez‐Erazo et al. 2022; J. Wang et al. 2023; Najari et al. 2024).

**TABLE 5:** Applications of complex fat replacers in frozen desserts.

Types	Sources	Applications	Suggested replacement ratio (%)	Functions	References
Protein–polysaccharide	SPI‐cellulose nanofiber	Milk ice cream	10 (with SPI‐cellulose nanofiber (7:1))	Increase viscosity, decrease melting rate and adhesiveness, achieve hardness, springiness, cohesiveness, and resilience similar to the full‐fat control	(Sun et al. 2015)
	SPI‐BCNs	Milk ice cream	≈48 (with 20% SPI‐BCNs (20:1))	Increase thermal stability, emulsifying capacity, viscosity, and viscoelasticity, provide creamy texture	(Guo et al. 2018)
	Microparticulated soy protein hydrolysates with 5% degree of hydrolysis (MSPH5)‐XG	Milk ice cream	50 (with 10% MSPH5‐XG (24:1))	Achieve appearance, taste, and texture comparable to the full‐fat control	(Liu et al. 2018)
	WPC‐Balangu Shirazi seed gum (BSSG)	Milk ice cream	96 (with 2.9% WPC‐BSSG (25:4) or 5.4% WPC‐BSSG (25:2))	Increase apparent viscosity, hardness, creaminess, and sensory acceptance, decrease overrun, melting rate, and coarseness	(Poursani et al. 2021)
Polysaccaride–polysaccaride	GG‐BSG	Vanilla ice cream	75 (with 0.55% GG‐BSG (1:1))	Provide excellent creaminess and satisfactory rheological properties	(Javidi et al. 2016)
Protein–lipid	SPI with HHP treatment (HSPI)‐water‐soluble phospholipid (PLW)	Soybean ice cream	≈50 (with 2.6% HSPI‐PLW (1000:3))	Increase the nitrogen soluble index (NSI), emulsifying activity, emulsion stability, foaming, and bubble stability, improve expansion, melting rate, and sensory acceptability, achieve overall qualities comparable to the full‐fat control	(Yan et al. 2022)
Polysaccharide–lipid	Fantesk™ (waxy maize starch‐butter)	Milk ice cream	≈86 (with 3.4% waxy maize starch‐butter (5:2))	Increase viscosity and elasticity, achieve overrun and melting behavior similar to the full‐fat control	(Byars 2002)
Lipid–lipid	Polysorbate 80 (PS80)‐mono‐ and diglycerides (MDG)	Vanilla ice cream	80 (with 0.17% or 0.22% PS80‐MDG (4:1))	Increase consistency of viscosity, whipping ability, and stability to heat shock, decrease ice crystal sizes, improve texture	(Baer et al. 1997)

#### 3.4.1 Binary Complexes

A binary combination of fat replacers has emerged as a promising approach for the development of reduced‐fat frozen desserts. These complexes can effectively mimic the textural and sensory attributes of fats, thereby maintaining or enhancing the quality of the final product. This section discusses three typical binary complexes used as fat replacers in frozen desserts: protein–polysaccharide, protein–lipid, and polysaccharide–lipid complexes, focusing on their mechanisms and applications.

**FIGURE 5:**
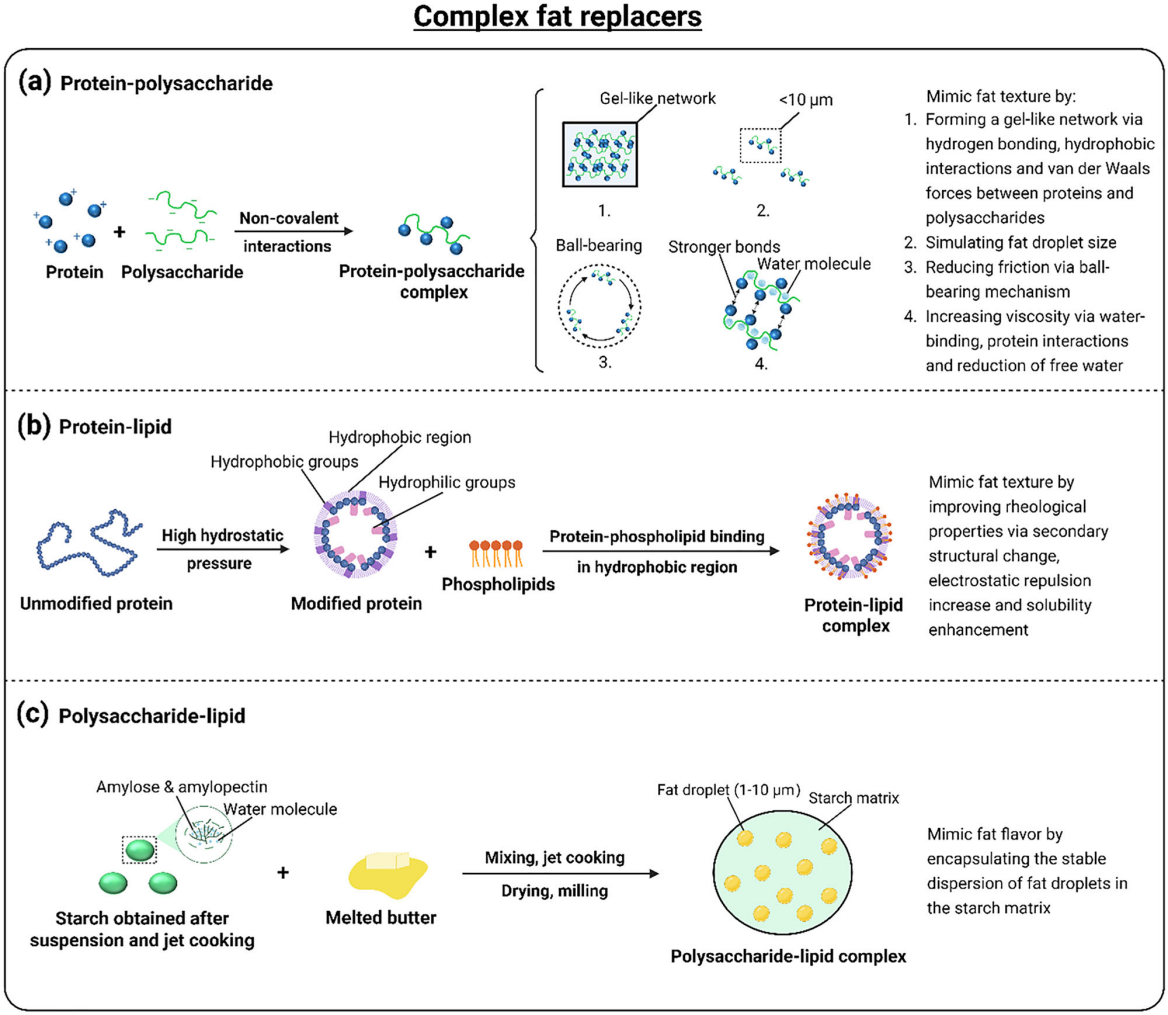
Schematics showing the mechanism of complex fat replaces in frozen desserts: (a) protein–polysaccharide complexes; (b) protein–lipid complexes; (c) polysaccharide–lipid complexes. *Source*: Created with BioRender.com.

Protein–polysaccharide complexes (Figure 5a) function as effective fat replacers in frozen desserts through four mechanisms that collectively mimic the texture and sensory attributes of fat. First, these complexes form a gel‐like network through noncovalent interactions, such as hydrogen bonding, hydrophobic interactions, and van der Waals forces, between proteins and polysaccharides. This network structure provides a structural framework that replicates the mouthfeel and stability typically associated with fats (Guo et al. 2018; H. Li et al. 2022; Han et al. 2023). For example, Sun et al. (2015) used SPI‐cellulose nanofiber gel for fat replacement, with 10% replacement achieving the desired outcomes of fat reduction, lower caloric content, enhanced antimelting properties, and comparable textural characteristics. Similarly, Guo et al. (2018) demonstrated that incorporating a 20% mixture of SPI and BC nanofibers (BCNs) as a protein–polysaccharide complex improved the melting rate, textural attributes, and sensory properties. Additionally, these complexes can simulate fat droplet sizes by creating microstructures smaller than 10 µm, contributing to a smoother texture (Liu et al. 2018). Liu et al. (2018) explored the use of microparticulated soy protein hydrolysate (MSPH)‐XG as a fat replacer for ice creams. They reported that a 50% fat‐replaced ice cream using MSPH with a hydrolysis degree of 5 (MSPH5)‐XG at a ratio of 24:1 had an appearance, taste, and texture similar to those of 10% full‐fat ice cream. Furthermore, the complex can reduce friction through a ball‐bearing mechanism and increase viscosity by binding water and reducing free water content, thereby enhancing the mouthfeel and stability of the frozen desserts (Poursani et al. 2021; Bourouis et al. 2023). Poursani et al. (2021) investigated the effects of replacing fat with WPC‐Balangu Shirazi seed gum (BSSG) at different concentrations on the rheological, physical, and sensory properties of nonfat ice cream (0.4% fat) compared to a full‐fat sample (10% fat). The results showed that increasing the concentration of WPC‐BSSG improved the apparent viscosity, hardness, creaminess, and sensory acceptance of nonfat ice cream, while decreasing the melting rate and coarseness of the ice cream.

Additionally, protein–lipid complexes (Figure 5b) rely on the structural modification of proteins to enhance their fat‐mimicking properties. Under HHP, proteins undergo conformational changes, exposing hydrophobic regions that bind to phospholipids (PL) (Yan et al. 2023). This binding creates a stable complex that improves rheological properties through secondary structural changes and increased electrostatic repulsion. The resulting complexes enhance solubility and provide a fat‐like texture by mimicking the flow and mouthfeel characteristics of fat (Han et al. 2021; Waghule et al. 2022; Chen et al. 2023). Yan et al. (2022) used HHP‐modified SPI (HSPI) combined with water‐soluble PL (PLW) as a fat replacer in ice cream. The results showed that HSPI‐PLW improved the functional properties of SPI, with a 163.40% increase in the nitrogen solubility index (NSI), 74.98% and 25.30% increases in emulsifying activity and stability, respectively, and 233.33% and 95.02% increases in foaming capacity and stability, respectively, compared to unmodified SPI. In addition, ice cream made with HSPI‐PLW exhibited a better expansion rate, lower melting rate, and acceptable sensory qualities similar to those of milk ice cream, demonstrating the potential of HSPI‐PLW as a fat replacer in low‐fat vegetable ice cream.

Finally, polysaccharide–lipid complexes (Figure 5c) mimic the fat flavor in frozen desserts by encapsulating fat droplets within a starch matrix (Aguilera 2019; Qazi et al. 2024). Starch obtained through suspension and jet cooking was mixed with melted butter, followed by jet cooking, drying and milling (Fanta and Eskins 1995; Byars 2002). The resulting complex stabilizes fat droplets (1–10 µm) within the starch matrix, creating a dispersion that mimics the flavor release of fats (Aguilera 2019). Byars (2002) compared the rheological properties of soft‐serve ice creams made with Fantesk™, a waxy maize starch–butter complex, to a commercial product. Fantesk™ was used at levels of 0.5–1.1 wt% butter in the ice cream mix. Despite its lower fat content, the Fantesk™‐based ice cream exhibited overrun and rheological properties similar to those of commercial products. Overall, these complexes offer promising applications for creating reduced‐fat frozen desserts with physicochemical and sensory properties similar to those of full‐fat products.

In summary, binary complexes, including protein–polysaccharide, protein–lipid, and polysaccharide–lipid complexes, have potential as fat replacers in frozen desserts. These complexes effectively mimic the textural and sensory attributes of fats through various mechanisms, such as the formation of gel‐like networks, enhancement of solubility, and encapsulation of fat droplets. The application of these complexes not only reduces fat content but also maintains or even enhances the quality of the final product.

#### 3.4.2 Ternary Complexes

In addition to binary complexes, ternary complexes have been explored as novel fat replacers in frozen desserts. Savio et al. (2018) used a formulation incorporating up to 5% (m/V) soybean protein concentrate (SPC), 14% (v/v) soybean hydrosoluble extract, and 15% (w/w) hydrogenated vegetable fat as a milk replacer for producing soybean gelato. This approach led to improvements in viscosity, non‐Newtonian flow behavior and protein stability. Another evident example of ternary complex fat replacement was reported by Nooshkam et al. (2023). In their study, a foam derived from a ternary complex comprising WPI, sodium alginate (SA), and licorice root extract served as an effective fat and sugar replacer in vanilla ice cream. At 25% replacement, the foam exhibited textural and sensory properties comparable to those of the control. Collectively, these findings demonstrate the potential of ternary complexes as innovative fat replacers in frozen dessert formulation.

## 4 Challenges and Strategies of Fat Replacers in Frozen Desserts

The use of fat replacers in frozen desserts is a critical strategy for developing healthier and reduced‐fat alternatives. However, the integration of fat replacers presents unique challenges that must be addressed to ensure desirable physicochemical and sensory properties of the final products. Protein‐, carbohydrate‐, lipid‐based, and complex fat replacers offer distinct advantages but also introduce specific challenges related to texture, flavor, color, and structural stability (Table 6). This section aims to provide a comprehensive overview of the challenges associated with these different types of fat replacers in frozen desserts and discuss the strategies developed to mitigate these issues, thereby enhancing the quality and consumer acceptance of reduced‐fat frozen desserts.

**TABLE 6:** Challenges and potential strategies of fat replacers in frozen desserts.

Types of fat replacers	Sources	Challenges	Strategies	References
Protein‐based	SPI	Provide soy‐associated off‐flavor, dark color, chalky or gritty texture, and low creaminess score	Decrease SPI concentration or add masking flavors such as chocolate	(Friedeck et al. 2003; Teh et al. 2005)
	PPI	100% PPI reduce smoothness, increase dark color, unfavorable taste, and odor	Decrease PPI concentration to 6% or 12%, or use spray‐ or freeze‐drying of PPI microparticulates	(Guler‐Akin et al. 2021; Tanger et al. 2021)
Carbohydrate‐based	CA‐treated SPS	2% CA‐treated SPS reduce hardness, lower sensory‐related scores	Decrease the concentration of CA‐treated SPS to 1%	(Surendra Babu et al. 2018)
	APS	Reduce viscosity, smoothness, mouthcoating properties, and consumer acceptance in ice cream with <5% fat	Change the source of modified starch and enhance the training of panelists, or combine starch with hydrocolloids	(Aime et al. 2001; M. Ma et al. 2024)
	Tapioca dextrin (TD)	Decrease creaminess, increase coarseness, wateriness, and chalkiness within 140 days of storage	Introduce micro‐gel particles or modify TD to create tiny gel particles (<5 µm)	(Specter and Setser 1994)
	Litesse^®^	Provide bitter taste, strong corn syrup flavor, and aftertaste, increase yellowness perceived as undesirable	Blend PD with MD	(Roland et al. 1999)
	GG	Interfere with sensory and nutritional properties, reduce protein efficacy and consumer acceptance, disrupt lipid utilization	Use partially hydrolyzed GG (PHGG)	(Mudgil et al. 2014)
	Rigid polysaccharides (XG and ι‐carrageenan (ι‐C))	Provide coldness and grittiness, form a gel network in serum phase, reduce air incorporation	Use flexible polysaccharides (LBG or GG) for desirable air incorporation, softness, and creaminess	(Liu et al. 2023)
Carbohydrate‐based	λ‐Carrageenan (λ‐C)	Decrease smoothness, increase thickness	Add a suitable level of inulin for better mouthfeel	(Brennan and Tudorica 2008; Bayarri et al. 2010)
	Oat and apple fibers	Increase sandiness and frozen water percentage, promote early ice nucleation and crystallization onset	Increase the amount of fibers dissolved in the mixture to enhance their solubility	(Soukoulis et al. 2009; Tolve et al. 2024)
	Orange fiber	Have bitter taste	Add a pretreatment to reduce compounds responsible for the bitterness in the orange	(de Moraes Crizel et al. 2013)
Lipid‐based	Oleogels prepared with EC	Decrease fat partial coalescence, network strength, and crystallinity, increase melting rate	Increase the viscosity of EC from cP10 to cP20	(Munk et al. 2018; N. Liu et al. 2024)
Complex	MWP (Simplesse^®^ 500)‐PD	Decrease creaminess and smoothness, increase melting rate and chalkiness compared to full‐fat control	Change PD to gums such as XG	(Prindiville et al. 2000; Regand and Goff 2003)
	Microcrystalline cellulose (MCC)‐GG	Exhibit high viscosity, low overrun, and limited whipping ability	Optimize milk fat, protein, and carbohydrate levels, use more than one fat replacer	(Adapa et al. 2000)
	Saturated MGs‐PS80	Decrease large fat aggregates and shape retention ability, increase melting rate at 0.2% and 0.3% dosage	Replace saturated MGs‐PS80 with unsaturated MGs	(Lee et al. 2018)

Protein‐based fat replacers in frozen desserts present unique challenges and strategies that must be carefully considered to achieve desirable sensory properties. SPI is a popular choice for fat replacement because of its high‐protein content; however, it often introduces soy‐associated off‐flavors such as green/grassy and doughy/fatty notes, dark color, chalky or gritty texture, and lower creaminess scores (Friedeck et al. 2003; Teh et al. 2005). These challenges can be mitigated by reducing the SPI concentration or adding masking flavors such as chocolate. Friedeck et al. (2003) found that incorporating chocolate flavor can effectively mask the undesirable taste of SPI while enhancing its overall acceptability. Similarly, PPI, another promising alternative, poses challenges when used as a fat replacer in frozen desserts. At higher concentrations (100%), PPI reduces smoothness, increases dark color, and imparts an unfavorable taste and odor, which negatively affect sensory perceptions (Guler‐Akin et al. 2021; Tanger et al. 2021). To address these issues, researchers recommend decreasing the PPI concentration to 6% or 12% (Guler‐Akin et al. 2021) or using spray‐ or freeze‐drying techniques to create microparticulates (Tanger et al. 2021) that improve textural attributes without compromising sensory quality. Both SPI and PPI positively contribute to physical properties such as viscosity and melting resistance; however, their impact on sensory characteristics necessitates strategic formulation adjustments to ensure consumer acceptance. Therefore, optimizing protein concentration and employing complementary ingredients or processing methods are essential for developing frozen desserts with enhanced nutritional profiles while maintaining an acceptable taste and texture.

Carbohydrate‐based fat replacers have gained attention in the development of reduced‐fat frozen desserts because of their ability to mimic fat sensory properties. However, several challenges have been identified in their application. For instance, the use of CA‐treated SPS as a fat replacer has been found to reduce hardness and lower sensory‐related scores when used at a 2% concentration (Surendra Babu et al. 2018). To address this issue, a decrease in the concentration of CA‐treated SPS to 1% has been proposed as an effective strategy. Similarly, GG, another commonly used carbohydrate‐based fat replacer, can interfere with sensory and nutritional properties, reduce protein efficacy and consumer acceptance, and disrupt lipid utilization (Mudgil et al. 2014). The use of partially hydrolyzed GG (PHGG) has been suggested as a solution to these challenges in food products. Moreover, rigid polysaccharides such as XG and ι‐C tend to provide coldness and grittiness, form a gel network in the serum phase, and reduce air incorporation (Liu et al. 2023). In contrast, flexible polysaccharides, such as LBG and GG, are more effective in achieving desirable air incorporation, softness, and creaminess. Finally, orange fiber can impart a bitter taste to products when used as a fat replacer (de Moraes Crizel et al. 2013). A potential solution to this problem is to implement a pretreatment process to reduce the compounds responsible for bitterness in orange fibers. Overall, these strategies aim to enhance the sensory quality and consumer acceptability of reduced‐fat frozen desserts while maintaining their nutritional value.

Lipid‐based fat replacers, such as oleogels prepared with EC, present specific challenges and strategies for their use in frozen desserts. One of the primary challenges with EC‐based oleogels is their tendency to decrease fat partial coalescence, network strength, and crystallinity, while simultaneously increasing the melting rate of desserts (Munk et al. 2018; N. Liu et al. 2024). These issues can lead to a less stable and undesirable texture in the final product. To address these challenges, one effective strategy is to increase the viscosity of the EC from cP10 to cP20, which has been shown to enhance the resistance to coalescence and improve the overall stability of fat globules (Munk et al. 2018). This approach helps create a stronger fat network, thereby improving the textural properties and structural integrity of frozen desserts. By optimizing the viscosity of the EC, the negative effects on fat partial coalescence and network strength can be mitigated, leading to a more satisfactory product.

Complex fat replacers also face distinct challenges that require tailored solutions to achieve the desirable sensory and structural properties of final products. For instance, the combination of MWP (Simplesse^®^ 500) with PD as a fat replacer results in decreased creaminess and smoothness, increased melting rate, and a sensation described as “chalkiness” compared to that of the full‐fat control (Prindiville et al. 2000). Chalkiness is a sensory attribute characterized by a dry, fine, powdery particulate sensation, often perceived when small particles are present in the food matrix (K. K. Ma et al. 2024). To address these issues, replacing PD with gums such as XG has been suggested as an effective strategy, as it improves meltdown resistance and enhances textural stability (Regand and Goff 2003). Another complex fat replacer system involves MCC combined with GG, which exhibits high viscosity, low overrun, and limited whipping ability. These limitations can be mitigated by optimizing the levels of milk fat, protein, and carbohydrates or by using more than one fat replacer in the formulation (Adapa et al. 2000). Furthermore, saturated MGs blended with PS80 reduced large fat aggregates and shape retention ability, while increasing the melting rate at 0.2% and 0.3% dosages. A potential solution is to replace saturated MGs‐PS80 blends with unsaturated MGs, which promote greater fat agglomeration and improve meltdown resistance and heat shock stability (Lee et al. 2018). Collectively, these findings highlight the importance of selecting appropriate combinations and concentrations of ingredients to overcome the challenges associated with complex fat replacers in frozen desserts, thereby ensuring both sensory appeal and structural integrity.

## 5 Conclusions and Future Prospectives

Frozen desserts are complex systems that rely on fat to achieve beneficial physicochemical and sensory attributes. However, the health risks associated with excessive fat consumption in frozen desserts have driven the development and application of fat replacers, which are categorized into protein‐, carbohydrate‐, lipid‐based, and complex types. This review analyzes the roles of fat and examines the mechanisms, effects, associated challenges, and strategies for using fat replacers in various types of frozen desserts. Fat replacers can mimic the physicochemical and sensory properties of natural fats in frozen desserts. Through mechanisms such as network formation, droplet size and shape simulation, friction reduction, viscosity increase, water retention, and flavor encapsulation, fat replacers contribute to fat‐like qualities while often reducing calorie content. Understanding these mechanisms can facilitate the development of new fat replacers with similar properties. Although many fat replacers can mimic the fat properties in frozen desserts, they still exhibit significant differences from natural fats, often resulting in undesirable textural and sensory changes. Therefore, achieving an appropriate balance of the modification, combination, and proportion of fat replacers is essential.

In the future, several research directions should be explored. First, the long‐term effects of fat replacers on human nutrition and gastrointestinal physiology should be studied. This ensures safe consumption and minimizes side effects. Second, more efforts are needed to investigate oral tribology and novel processing techniques, such as the combination of high‐pressure homogenization and ultrasound, to accurately understand how fat replacers contribute to the desirable characteristics of full‐fat products. These advancements are critical for improving consumer acceptance of these products. Furthermore, future research should investigate sustainable ingredients with low environmental footprints, including microbial proteins, as potential fat replacers in frozen desserts to meet the sustainable development goals outlined by the United Nations. By exploring these research directions, the food industry can continuously innovate and offer healthier and more satisfying reduced‐fat foods.

## Author Contributions

**Zhaoyi Tang**: conceptualization, writing–original draft, writing–review and editing, investigation, methodology, validation, software, formal analysis, visualization, data curation. **Shuyue Yang**: visualization, data curation, investigation, writing–review and editing. **Weitian Li**: investigation, data curation. **Jinhui Chang**: conceptualization, supervision, project administration, funding acquisition, resources, writing–review and editing.

## Conflicts of Interest

The authors declare no conflict of interest.
